# Cardiovascular Involvement in Tuberculosis Patients Treated in Southern Africa

**DOI:** 10.1016/j.jacadv.2024.101427

**Published:** 2024-12-05

**Authors:** Daryoush Samim, Guy Muula, Nicolas Banholzer, Douglas Chibomba, Sihle Xulu, Carolyn Bolton, Denise Evans, Lisa Perrig, Stefano De Marchi, Gunar Günther, Matthias Egger, Thomas Pilgrim, Lukas Fenner, IeDEA Southern Africa (IeDEA-SA)

**Affiliations:** a Department of Cardiology, Inselspital, Bern University Hospital, University of Bern, Bern, Switzerland; b Centre for Infectious Disease Research in Zambia (CIDRZ), Lusaka, Zambia; c Institute of Social and Preventive Medicine (ISPM), University of Bern, Bern, Switzerland; d University Teaching Hospital, Department of Internal Medicine, Lusaka, Zambia; e Department of Cardiology, Helen Joseph Clinic, Johannesburg, Republic of South Africa; f Health Economics and Epidemiology Research Office, Faculty of Health Sciences, University of the Witwatersrand, Johannesburg, South Africa; g Department of Pulmonology and Allergology, Inselspital, University Hospital of Bern, Bern, Switzerland; h Centre for Infectious Disease Epidemiology and Research, University of Cape Town, Cape Town, Republic of South Africa; i Population Health Sciences, Bristol Medical School, University of Bristol, Bristol, UK

**Keywords:** constrictive pericarditis, echocardiography, HIV, pericardial effusion, tuberculosis

## Abstract

**Background:**

Tuberculosis (TB) is the leading cause of death among people with HIV and a major global health challenge. Subclinical cardiovascular manifestations of TB are poorly documented in high TB and HIV burden countries.

**Objectives:**

The purpose of this study was to quantify the prevalence of cardiovascular involvement in TB patients and investigate changes after completion of anti-TB treatment.

**Methods:**

HIV-positive and HIV-negative patients diagnosed with pulmonary TB between October 2022 and November 2023 were enrolled from 2 tertiary care hospitals in Zambia and South Africa. Standardized transthoracic echocardiography (TTE) was conducted at TB diagnosis and after 6 months of anti-TB treatment. Cross-sectional and longitudinal analyses assessed pericardial effusion, thickening, or calcification, with and without signs of pericardial constriction.

**Results:**

A total of 286 TB patients (218 [76%] men, 109 [38%] people with HIV, median age 35 years) underwent TTE at TB diagnosis, of whom 105 participants had a second TTE after completion of treatment. At TB diagnosis, 134 (47%) had pericardial effusions, 86 (30%) thickening, 7 (2%) calcifications, 103 (42%) signs of constriction, and 13 (12%) had definite diagnosis of constriction. After TB treatment, pericardial effusions (47% vs 16%, *P* < 0.001) and pericardial thickenings (30% vs 15%, *P* = 0.002) became less prevalent. Pericardial calcifications (2% vs 1%, *P* = 0.4), signs of constrictions (42% vs 38%, *P* = 0.4), and definite diagnosis of constriction (12% vs 14%, *P* = 0.8) were similar.

**Conclusions:**

Cardiac involvement is frequent in newly diagnosed TB patients. Early pericardial changes may be reversed with anti-TB treatment. Echocardiographic screening facilitates early detection and timely management of cardiovascular involvement in TB patients.

Tuberculosis (TB) remains a major public health challenge worldwide and has received renewed attention with the emergence of multi drug-resistant *Mycobacterium tuberculosis*. TB is the leading cause of death among people living with HIV^1^ and accounts for 1.4 million deaths every year.^1^

Populations in Africa are undergoing both an epidemiological and demographic transition with increased survival from childhood to adulthood.^2^ Noncommunicable diseases are projected to outpace communicable diseases within the current decade.^3^ Although ischemic heart disease is rapidly becoming a significant contributor, to date, heart disease in Africa remains dominated by nonischemic etiologies such as hypertensive heart disease and infectious pathologies, particularly rheumatic heart disease and TB-related conditions.^2^, ^3^, ^4^ Advanced stages of pulmonary TB can result in pulmonary hypertension, eventually leading to right heart failure.^5^ In addition, direct cardiac involvement of TB is a serious extra-pulmonary complication affecting the pericardium, the myocardium, or the aorta.^3^ Manifestations of cardiac TB range from pericardial effusion and morphological changes of the pericardium to constrictive pericarditis or myocarditis. In addition, the toxicity associated with anti-TB drugs may contribute to the development of heart failure, further increasing morbidity and mortality.^6^

In historical studies, TB pericarditis was found in approximately 1% of all autopsied cases of TB and in 1% to 2% of instances of pulmonary TB.^7^ TB is the predominant cause of constrictive pericarditis in Africa, accounting for up to 10% of heart failure hospitalizations.^8^^,^^9^ Effective strategies are needed to reduce the burden of TB and prevent extra-pulmonary complications. Timely detection of early, potentially reversible stages of cardiac involvement and early initiation of anti-TB treatment may prevent progression to irreversible stages of cardiac damage.^4^ However, little is known about the contemporary prevalence of cardiovascular involvement in patients with a new diagnosis of pulmonary TB, and the responsiveness to anti-TB treatment. In a prospective cohort study in Zambia and South Africa, we aimed to determine the prevalence of different stages of cardiovascular involvement in HIV-positive and HIV-negative individuals diagnosed with TB, and to assess the changes of cardiovascular manifestations of TB in response to anti-TB treatment.

## Methods

### Study setting and study design

Using a prospective, noninterventional cohort study, we consecutively enrolled patients recently diagnosed with pulmonary TB living with and without HIV, and with and without drug-resistant TB at 2 antiretroviral therapy (ART) clinics in the International epidemiology Databases to Evaluate AIDS in Southern Africa (IeDEA-SA) region and affiliated TB clinics:^10^ Centre for Infectious Disease Research in Zambia (CIDRZ), Lusaka, Zambia; Themba Lethu Clinic, Helen Joseph Hospital, Johannesburg, Republic of South Africa.^11^ We prospectively included individuals aged ≥15 years diagnosed with active pulmonary TB that was either bacteriologically confirmed (GeneXpert Ultra or positive lipoarabinomannan urine tests) or clinically diagnosed based on clinical symptoms and chest x-ray results. TB diagnosis and treatment were managed according to the existing local standard of care.

### Eligibility criteria

To be eligible, an individual with a diagnosis of pulmonary TB had to meet the following inclusion criteria: 1) age ≥15 years; 2) documented HIV test or willingness to be tested; and 3) written informed consent (additional written assent from participants 15-18 years of age). Individuals meeting any of the following criteria were excluded from participation: 1) >1 week of anti-TB treatment within the prior 30 days except anti-TB preventive therapy; 2) plans to follow-up for TB care at a site distant from the enrollment site; 3) cognitive impairment; 4) imprisonment; 5) absence of pulmonary TB; and 6) missing baseline visit.

### Study procedures

This cohort of TB patients was followed for up to 18 months after starting anti-TB treatment.^11^ Recruitment started in October 2022. We performed transthoracic echocardiography (TTE) at the time of pulmonary TB diagnosis and treatment initiation (baseline), at the end of anti-TB treatment (after 6 months), and 6 months after the end of anti-TB treatment. The time points of TTEs are shown in Supplemental Figure 1. For this study, we included all patients recruited and followed up by the end of November 2023. TTEs after 6 months of completed treatment were only analyzed when there were prior pericardial abnormalities.

### Data collection

We performed TTEs at the cardiology department associated with the ART/TB clinics by trained physicians or echocardiographic technicians (Cardiology Department, University Teaching Hospital, Lusaka, Zambia; Cardiology Department, Helen Joseph Clinic, Johannesburg, Republic of South Africa). The TTEs were performed according to a standardized procedure as previously described ^7^ and summarized in Supplemental Text 1. At baseline, we collected epidemiological and clinical variables.

### Echocardiographic analysis

Pericardial thickening, pericardial fibrin strands or echo densities, and pericardial calcification were visually assessed.^12^ If present, pericardial effusion was categorized as mild (end-diastolic <10 mm), moderate (end-diastolic 10-20 mm), or large (end-diastolic >20 mm).

The following isolated signs of constriction were considered: E/A ratio of transmitral flow velocity >1.5 with deceleration time < 160 and inferior vena cava (IVC) > 21 mm or <50% IVC-respiratory variability, variation in mitral inflow E velocity (E-wave decrease >25% during inspiration), respirophasic septal shift and/or diastolic septal bounce, E' medial/lateral >0.9 (annulus reversus), diastolic hepatic vein expiratory reversal/forward flow velocity ≥0.8.^13^^,^^14^ E' medial ≥9 cm/s was not counted as an isolated sign of constriction since it is commonly elevated in healthy young individuals.^14^

Definite constrictive pericarditis was defined as E/A>0.8 and IVC>21 mm and/or <50% respiratory variability with respirophasic septal shift (and/or diastolic septal bounce) AND: E' medial ≥9 cm/s, OR E' medial between 6 and 9 cm/s with E' medial/lateral >0.9 (annulus reversus), OR diastolic hepatic vein expiratory reversal/forward flow velocity ≥0.8.^13^^,^^15^ Echocardiography diagnostic criteria algorithm for constrictive pericarditis is shown in Supplemental Figure 2.

### Study outcomes

An experienced echocardiographer from the department of cardiology at Bern University Hospital (DS), who was blinded to all clinical parameters, assessed every TTE for pericardial effusion, pericardial thickening, pericardial fibrin strands or echo densities, pericardial calcification, signs of constriction and constrictive pericarditis, as well as left ventricular (LV) systolic dysfunction and dilatation of the ascending aorta.

### Statistical analysis

The proportion of patients with pericardial abnormalities at TB diagnosis and end of TB treatment were compared using tests for equal proportions. Prevalence of pericardial abnormalities was compared considering all patients (cross-sectional analysis) and only patients with follow-up visits (paired analysis). As a sensitivity analysis, we imputed missing (not determinable) echocardiographic parameters assuming missing at random. Each echocardiographic parameter was imputed using multivariate imputation by chained equations considering other echocardiographic parameters and patient characteristics.^16^ Statistical comparison of pericardial abnormalities was performed on 100 imputed data sets and the estimation results were pooled using Rubin’s Rule.^17^ The association of epidemiological and clinical characteristics with pericardial abnormalities was estimated using univariable and multivariable logistic regression models with site-specific fixed effects. Associations were expressed as OR and stratified by time of visit. Estimation results were expressed as means and 95% CIs or *P* values. All analyses were performed in R, version 4.3.2.

### Ethical statement

The University of the Witwatersrand, Human Research Ethics Committee (no. M220141), the University of Zambia Biomedical Research Ethics Committee (no. 2538-2022), and the Cantonal Ethics Committee of Bern, Switzerland approved the project (no. PB_2016-00273). All study participants provided written informed consent.

## Results

A total of 286 TB patients were recruited between October 2022 and November 2023 in Zambia (n = 235, 82%) and South Africa (n = 51, 18%); median follow-up time 197 days (IQR: 186 to 230 days). Epidemiological and clinical characteristics, as well as laboratory parameters are summarized in Table 1. The median age was 35 (IQR: 28-42) years old. One-third (96 patients, 34%) of patients were underweight (body mass index <18 kg/m^2^). One-quarter of the patients (69, 24%) were relapse TB cases, 20 (7%) were TB drug-resistant patients (at least rifampicin-resistant), and 59 (21%) had cavitating disease. At the end of treatment, 105 (37%) were re-examined, and 6 months later, another 12 patients underwent echocardiography. 7 patients died during treatment, 4 of them were living with HIV.

**Table 1:** Epidemiological Characteristics, Clinical Characteristics, and Laboratory Blood Test Results in TB Patients at the Time of Diagnosis

Variable	Total (N = 286) n (%)/Median (IQR)
Site	
South Africa	51 (18%)
Zambia	235 (82%)
Median age (IQR), years	35 (28-42)
Sex	
Male	218 (76%)
Female	68 (24%)
TB case	
Relapse	69 (24%)
New case	217 (76%)
Cavitary disease	
Yes	59 (21%)
No	227 (79%)
Anti-TB drug resistance (at least rifampicin-resistant)	
No	266 (93%)
Yes	20 (7%)
TB manifestation	
Pulmonary	285 (100%)
HIV infection status	
Negative	177 (62%)
Positive	109 (38%)
CD4 count <350/mm^3^	45 (41%)
CD4 count ≥350/mm^3^	38 (35%)
CD4 count unknown	26 (24%)
Median CD4 cell count (IQR), mm^3^	320 (134-494)
HIV viral load (copies/mL)	
Undetectable	17 (22%)
Detectable	60 (78%)
Median HIV-RNA viral load (IQR), copies/mL	232 (59-238859)
BMI (kg/m^2^)	19.1 (17.3-21.2)
Obese (BMI >30 kg/m^2^)	13 (5%)
Normal (18 kg/m^2^ ≤BMI ≤30 kg/m^2^)	169 (59%)
Underweight (BMI <18 kg/m^2^)	96 (34%)
Unknown	8 (3%)
Smoking	
Yes	71 (25%)
No	215 (75%)
Systolic blood pressure (mm Hg)	114 (104-126)
Diastolic blood pressure (mm Hg)	77 (69-86)
6 min walk-test distance (m)	368 (320-448)
Sit-to-stand test (repetitions per min)	16 (14-19)
Medical history of hypertension	
Yes	14 (5%)
No	270 (95%)
Hematological and inflammation parameters	
CRP (mg/l)	27.00 (8.00-87.50)
Neutrophils (G/I)	4.00 (2.67-5.80)
Lymphocytes (G/I)	1.40 (1.07-1.88)
Eosinophils (G/I)	0.05 (0.02-0.15)
Hemoglobin (g/dl)	11.90 (10.30-13.40)

Categorical variables as positive count (% of total count) and continuous variables as median and IQR.

BMI = body mass index; CRP = c-reactive protein; IQR = interquartile range; TB = tuberculosis.

### Echocardiographic findings and cardiovascular involvement of TB at TB diagnosis

**Figure 1:**
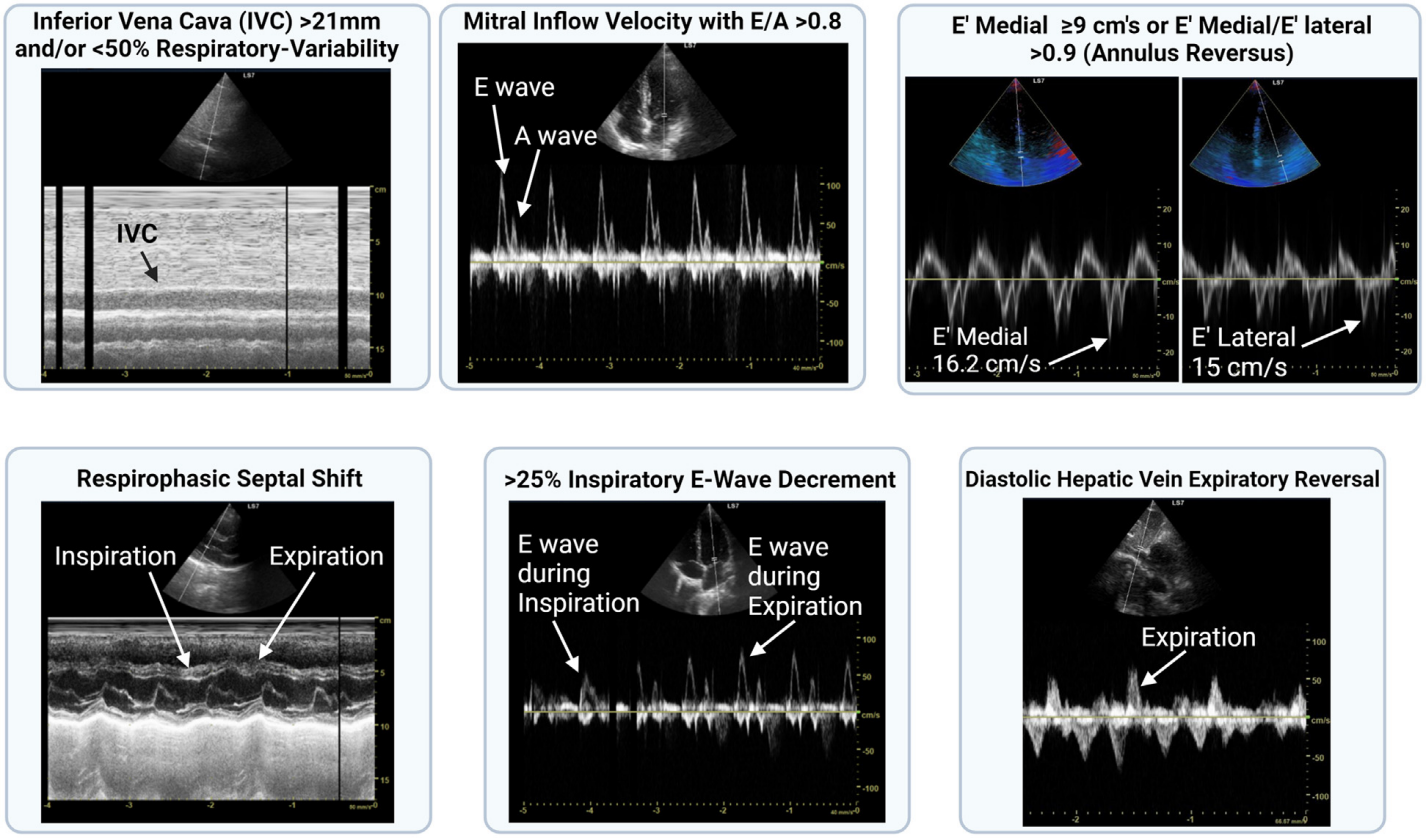
**Echocardiographic Findings of Pericardial Constriction** Various signs of constriction observed during the study.

Echocardiographic findings at TB diagnosis (baseline) and echocardiographic findings consistent with extrapulmonary manifestations of TB are summarized in Table 2 and shown in the Central Illustration. Overall, 190 (66%) of the patients had any pericardial abnormality at the start of diagnosis. Almost half the patients had pericardial effusion (134 of 286 patients, 47%) (Video 1), which was mild in a majority of patients (109, 81%). We found 8 patients with fibrin strands (Video 2) or echo densities (8 of 286, 3%). One-third of patients (86 of 286, 30%) had pericardial thickening, while the presence of pericardial calcification was rare (7 of 285, 2%). Signs of constriction were common (103 of 244, 42%); 13 out of 105 patients (12%) were found to have definite evidence of pericardial constriction (Figure 1 and Video 3). Any pericardial abnormalities at baseline were not significantly more frequent among patients who subsequently died (86% [6/7] vs 66% [184/279], *P* = 0.3).

**Table 2:** Echocardiography Results in TB Patients at the Time of Diagnosis and During Follow-Up

Variable	At TB Diagnosis (N = 286) n (%)/Median (IQR)	End of Anti-TB Treatment (N = 105) n (%)/Median (IQR)	Post Anti-TB Treatment (N = 12) n (%)/Median (IQR)
Pericardial effusion	134/286 (47%)	16/98 (16%)	0/12 (0)
Mild (end-diastolic <10 mm)	109 (81%)	14 (88%)	0 (0)
Moderate (end-diastolic 10-20 mm)	22 (16%)	2 (12%)	0 (0)
Large (end-diastolic >20 mm)	3 (2%)	0 (0)	0 (0)
Pericardial thickening	86/286 (30%)	15/102 (15%)	1/12 (8%)
Pericardial calcification	7/285 (2%)	1/100 (1%)	0/12 (0)
Presence of fibrin strands or pericardial echo densities	8/286 (3%)	0/88 (0)	0/9 (0)
Signs of constrictions	103/244 (42%)	33/88 (38%)	4/9 (44%)
E/A>1.5 with DT < 160 ms and IVC>21 mm and/or < 50% IVC-respiratory variability	40/246 (16%)	14/93 (15%)	1/8 (12%)
Variation in mitral inflow E velocity (E-wave decrease >25% during inspiration)	17/239 (7%)	2/92 (2%)	1/9 (11%)
Respirophasic septal shift and/or diastolic septal bounce	45/249 (18%)	6/97 (6%)	1/12 (8%)
E' medial/lateral >0.9 (annulus reversus)	11/83 (13%)	16/59 (27%)	4/11 (36%)
Diastolic hepatic vein expiratory reversal and/or forward flow velocity ≥0.8	1/286 (0)	8/105 (8%)	2/12 (17%)
Definite diagnosis of constriction	13/105 (12%)	9/65 (14%)	0/7 (0)
Abnormal LV geometry	129/266 (48%)	45/87 (52%)	0/0 (0)
Concentric remodeling	112 (87%)	43 (96%)	0 (0)
Concentric hypertrophy	11 (9%)	2 (4%)	0 (0)
Eccentric hypertrophy	6 (5%)	0 (0)	0 (0)
Aortic root dilatation	3/241 (1%)	0/86 (0)	0/0 (0)
Ascending aorta dilatation	3/230 (1%)	1/84 (1%)	0/0 (0)
Relevant aortic valve disease	4/270 (1%)	0/90 (0)	0/0 (0)
Aortic stenosis	0/4 (0)	0/0 (0)	0/0 (0)
Aortic regurgitation	4/4 (100%)	0/0 (0)	0/0 (0)
Relevant mitral valve disease	3/270 (1%)	1/89 (1%)	0/0 (0)
Mitral stenosis	0/3 (0)	0/1 (0)	0/0 (0)
Mitral regurgitation	3/3 (100%)	1/1 (100)	0/0 (0)
Relevant tricuspid valve disease	2/266 (1%)	0/83 (0)	0/0 (0)
Tricuspid stenosis	0/2 (0)	0/0 (0)	0/0 (0)
Tricuspid regurgitation	2/2 (100%)	0/0 (0)	0/0 (0)
LVEF (%, visually assessed)	60.0 (55.0-65.0)	60.0 (55.0-65.0)	-
LVEF (%, Simpson BP)	60.3 (56.3-65.0)	59.5 (56.5-65.0)	-
LV systolic dysfunction	3/283 (1%)	1/103 (1%)	0/0 (0)
Diastolic dysfunction	8/86 (9%)	3/57 (5%)	0/7 (0)
Grade I	2/8 (25%)	0/3	0/0
Grade II	1/8 (12.5%)	0/3	0/0
Grade III	0/8	0/3	0/0
Undefined	5/8 (62.5%)	3/3 (100%)	0/0
LV dilatation	2/262 (1%)	0/86 (0)	0/0 (0)
LA dilatation	44/266 (17%)	6/86 (7%)	3/9 (33%)
RV dilatation	15/240 (6%)	1/72 (1%)	0/0 (0)
RA dilatation	27/227 (12%)	7/71 (10%)	0/0 (0)
TAPSE (mm)	22.3 (19.5-25.0)	23.6 (21.0-26.0)	-
TV annulus DTI S' (cm/sec)	13.0 (12.0-14.7)	12.7 (11.6-14.0)	-
RV longitudinal dysfunction	11/264 (4%)	3/97 (3%)	0/0 (0)
RV FAC (%)	46.7 (42. −51.5)	44.4 (38.4-48.1)	-
RV global dysfunction	0/227 (0)	1/68 (1%)	0/0 (0)
RV/RA gradient (mm Hg)	21.1 (18.6-27.5)	22.6 (18.2-27.7)	16.0 (16.0-16.0)
Estimated central venous pressure (mm Hg)	10 (5-15)	10 (10-10)	10 (10-15)

Categorical variables as positive count (% of total count) and continuous variables as median and IQR. Participants for whom echocardiography results could not be determined were excluded.

LV = left ventricular; LVEF = left ventricular ejection fraction; LA = left atrial; RV = right ventricular; RA = right atrial; TAPSE = tricuspid annular plane systolic excursion; TV annulus DTI S’ = derived tricuspid lateral annular systolic velocity wave S’; IQR = interquartile range; FAC = fractional area change; TB = tuberculosis; IVC = inferior vena cava; DT = deceleration time.

**Central Illustration:**
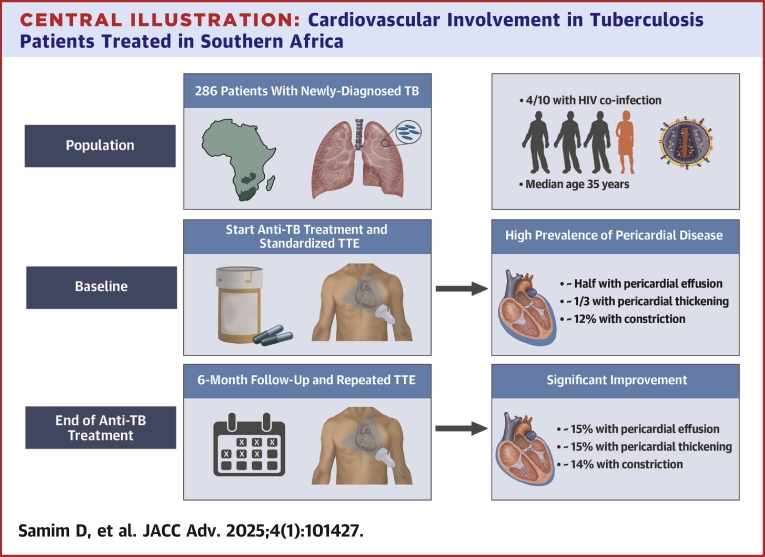
Cardiovascular Involvement in Tuberculosis Patients Treated in Southern Africa TTE = transthoracic echocardiography; TB = tuberculosis.

At baseline, 129 of 266 patients (48%) had abnormal LV geometry, 112 of whom (87%) had concentric LV remodeling. Two patients had LV dilatation (2 of 262 [1%]) and 3 patients (3 of 283, [1%]) had LV systolic dysfunction. Right ventricular (RV) dilatation was recorded in 15 (15 of 240 patients, 6%), and RV longitudinal dysfunction in 11 (11 of 264, 4%) patients, respectively. Median RV/RA pressure gradient was normal, although it could only be measured in 13% of patients due to the low prevalence of tricuspid regurgitation. More than mild aortic, mitral and tricuspid regurgitation were documented in 4 of 270 (1%), 3 of 270 (1%), and 2 of 266 (1%) patients, respectively. Aortic root dilatation (3/241) and dilatation of the ascending aorta (3/230) were documented in 1% for each.

### Findings after completion of anti-TB treatment and beyond

Echocardiographic follow-up was performed in 105 patients after completion of a 6-month course of anti-TB treatment (37%), and 12 patients underwent follow-up 6 months after the end of treatment (4%). Cardiac manifestations of TB at the end of anti-TB treatment and 6 months after completion are shown in Table 2. Proportions of pericardial abnormalities at baseline and end of anti-TB treatment are presented in Supplemental Figure 3, Panel A.

**Figure 2:**
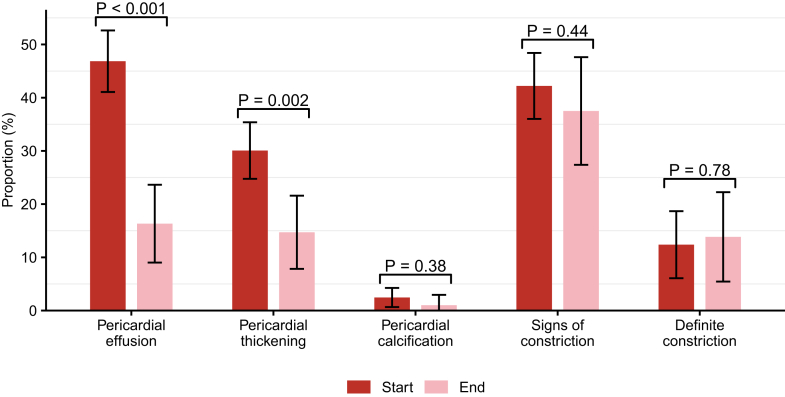
**Changes of Pericardial Pathologies Between Baseline and End of Anti-Tuberculosis Treatment** Proportion of patients with pericardial abnormalities in patients with follow-up visits (105 patients) at the start and at the end of anti-TB treatment. Shown is the mean (vertical bars) and standard deviation (error bars) of the proportion after data imputation. TB = tuberculosis.

Based on a complete case analysis of all patients (N = 286), we estimated a reduction in the proportion of pericardial effusions between TB diagnosis and end of anti-TB treatment (47% vs 16% *P* < 0.01) and in pericardial thickenings (30% vs 15%, *P* = 0.002) (Figure 2). The proportion of fibrin strands or pericardial echo densities (3% vs 0%, *P* = 0.11), pericardial calcifications (2% vs 1%, *P* = 0.38), signs of constrictions (42% vs 38%, *P* = 0.44), and definite diagnosis of constriction (12% vs 14%, *P* = 0.78) were comparable. Pooled results from the analysis of imputed data were similar (Supplemental Table 3, Panel B). Based on analyses restricted to patients with follow-up TTEs (N = 105), we found a higher prevalence of pericardial abnormalities at TB diagnosis and estimated greater reductions after anti-TB treatment (Supplemental Table 3, Panel C and D).

**Figure 3:**
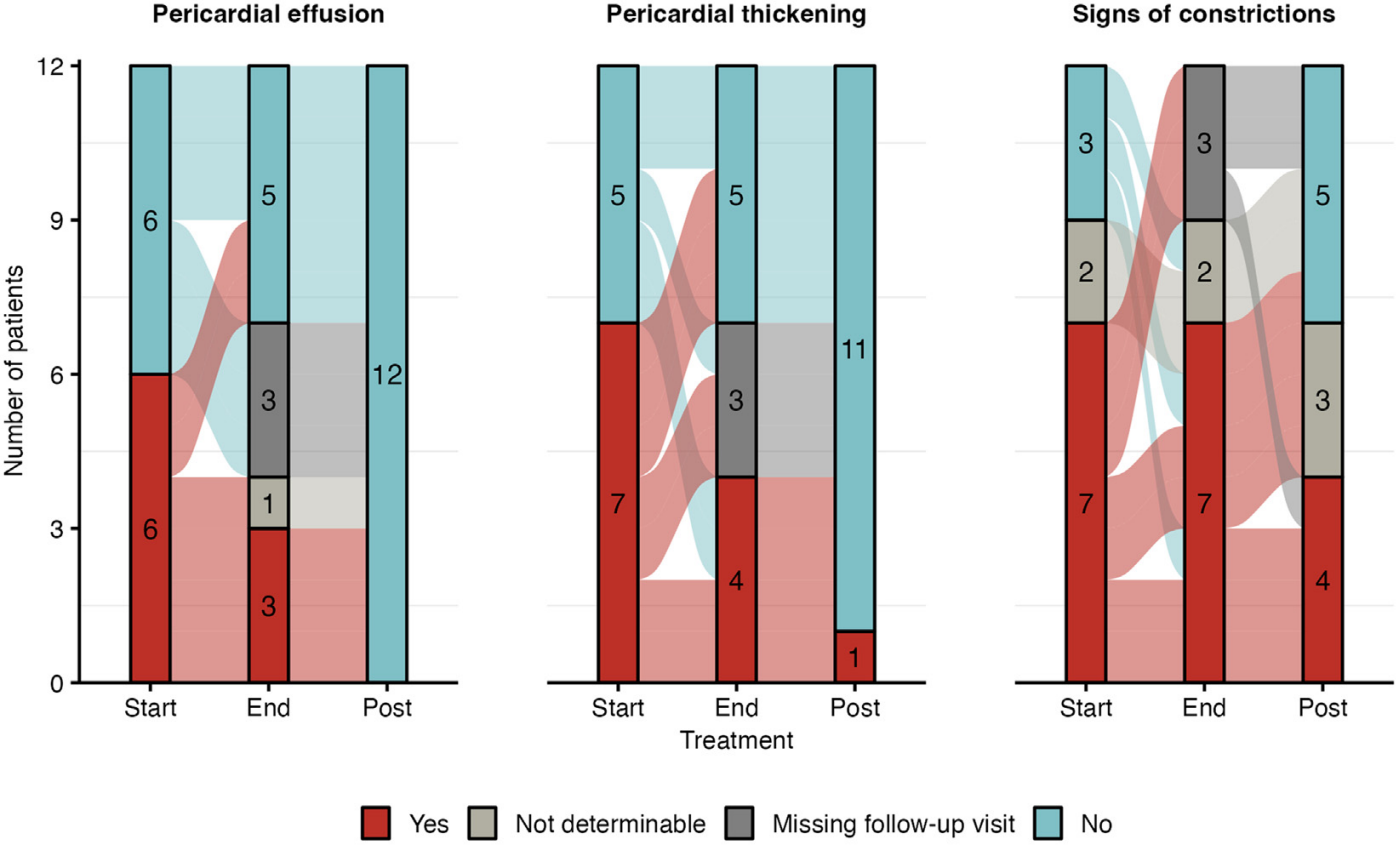
**Pericardial Developments Through Anti-Tuberculosis Treatment and Six Months After Completed Anti-Tuberculosis Treatment** Changes in pericardial abnormalities following anti-TB treatment in patients with post-treatment echocardiography images (12 patients). Abbreviation as in Figure 2.

In 12 patients with TTEs 6 months post end of TB treatment (12 patients), echocardiographic improvements continued post-treatment (Figure 3). Pericardial effusions and thickenings disappeared almost entirely, and signs of constrictions disappeared in 3 out of 7 patients.

### Prevalence and evolution of cardiac involvement in people with HIV

The prevalence of HIV coinfection was 38% (109 out of 286 patients). All patients with HIV were on ART. HIV viral load was detectable in 60 patients (78%), and 45 (41%) had low CD4 count (<350/mm^3^). Echocardiographic information according to HIV infection status at the start and end of anti-TB treatment is presented in Supplemental Tables 1 and 2. The findings in HIV-positive patients were consistent with the observations reported for the overall population.

### Predictors of regression of cardiac involvement

Pericardial abnormalities (pericardial effusions, pericardial thickenings, and signs of constrictions) at TB diagnosis and end of anti-TB treatment were not significantly associated with baseline patient characteristics (Supplemental Tables 4 and 5). Pericardial thickening at TB diagnosis tended to be more prevalent in HIV-positive patients (OR: 1.62; 95% CI: 0.95-2.75) and signs of constrictions at the end of anti-TB treatment tended to be less prevalent in relapse TB cases (OR: 0.38; 95% CI: 0.13-1.09). Associations of pericardial calcifications and definite diagnosis of constriction were not analyzed due to low prevalence.

## Discussion

The main findings of this cross-sectional and longitudinal analysis of patients with a diagnosis of pulmonary TB in Zambia and South Africa can be summarized as follows: 1) about 2-thirds of patients had early signs of cardiac involvement at the start of anti-TB treatment; 2) pericardial effusion and pericardial thickening were the most common echocardiographic manifestations; 3) constrictive pericarditis was detected in 12%, while any sign indicating constrictive hemodynamics was recorded in more than 40% of cases; and 4) early stages of pericardial involvement largely showed regression after completion of an anti-TB treatment, while constrictive pericarditis persisted throughout the course of anti-TB treatment. To our knowledge, this is the first study to analyze pericardial changes in pulmonary TB patients in 2 different sub-Saharan African study sites at baseline and during follow-up using standardized TTE protocols.

In Southern Africa, pericardial TB is the second most common form of extrapulmonary TB, second only to pleural disease.^9^ Pericardial TB often develops through lymphatic or hematogenous spread from a primary infection, typically in the lungs, or from distant secondary infections. Subclinical immunosuppression, which is not necessarily related to HIV infection, but may be associated with conditions such as malnutrition, may facilitate the spread of TB bacilli.^18^ In our cohort, over one-third of patients were malnourished. Evidence of simultaneous pulmonary and pericardial TB is limited due to underdiagnosis and lack of autopsy studies. Pericardial TB is often regarded as a paucibacillary condition, with immune-mediated inflammation playing a central role in its pathology^19^ Clinical presentations of TB pericarditis include acute pericarditis, pericardial effusion (leading to tamponade in extreme cases), effusive-constrictive pericarditis, or constrictive pericarditis, which describe a four-stage evolution starting with acute pericarditis, stage 1, the “dry stage,” the least common form seen.^20^ Stage 2 is effusive or effusive-constrictive pericarditis, the form most frequently seen, particularly in sub-Saharan Africa.^20^ Constrictive pericarditis with radiological and echocardiographic evidence of pericardial thickening and fibrinous fluid around the heart is stage 3.^20^ Finally, “pure” constrictive pericarditis with no residual fluid in the pericardium is stage 4.^20^

In North America and Europe, the estimated prevalence of pericardial effusion in the general population is 6% to 9%,^21^^,^^22^ while corresponding data from lower-income countries, where the leading underlying cause is TB, are lacking. Data from the Framingham cohort showed that the prevalence of pericardial effusion increases with age, from <1% in patients aged 20 to 30 years to over 15% in patients aged >80 years.^23^ In our study, which involved young patients with active pulmonary TB and signs of systemic inflammation from 2 countries in Southern Africa, the high prevalence at baseline of pericardial effusion (47%) and pericardial thickening (one-third of the cohort) sometimes accompanied by fibrin strands/echo densities supports the hypothesis of pericardial inflammation.

A recent study in the general population of Würzburg in Germany, a country with a low incidence of TB, found a prevalence of incidental pericardial effusion of 2.7% at baseline and 1.5% after a median follow-up of 34 months.^22^ In our study, after anti-TB treatment, the high prevalence of signs of pericardial inflammation decreased considerably, from 47% to 16% for pericardial effusion and 30% to 15% for pericardial thickening. Given the context of active TB with systemic inflammation at baseline, we postulate an antimicrobial and anti-inflammatory effect of anti-TB treatment.^24^^,^^25^ However, given the high proportion of small pericardial effusion, a physiological fluctuation cannot be definitively ruled out.

Stage 2, symptomatic pericardial effusion, has been found to be more frequent in patients with AIDS.^8^ However, a prospective multicenter cohort study conducted in Germany during the ART era has demonstrated that the prevalence of pericardial effusion is very low in HIV-positive outpatients under ART.^26^ *M tuberculosis* is the underlying cause in up to 70% of cases of pericardial effusion in HIV-positive patients.^27^ In our study, we found no association between pericardial effusion and HIV coinfection. In addition, we found no association between signs of constriction and HIV coinfection. Recent reports show that HIV-associated TB pericarditis is associated with a lower rate of progression to constriction.^28^, ^29^, ^30^, ^31^ However, patients with HIV-associated TB pericarditis have greater prevalence of myopericarditis and markedly increased mortality.^28^^,^^32^ In our study, pericardial abnormalities were not strongly associated with patient characteristics, including HIV coinfection. This supports the hypothesis that pericardial abnormalities observed in our study are mainly related to active TB. However, it is important to recall the observational nature of our study.

We observed a prevalence of constrictive TB pericarditis in our study that is higher than the 2% to 5% reported in previous studies in TB^8^. The relatively young age of our study population may contribute to the observed higher prevalence, as middle-aged (35-55 years) patients are at an increased risk of developing constriction following acute pericarditis.^33^ The predominance of men in our study population might also account for this higher prevalence.^33^^,^^34^ The causes of sex differences in pericardial inflammation are unknown, but testosterone seems to play a pro-inflammatory role in cardiac inflammation,^35^ whereas estrogens are known for their cardioprotective, anti-inflammatory effect.^36^^,^^37^

The typical incubation period of TB is 3 to 9 months.^38^ The delay between the initial pericardial inflammation and the onset of constriction (stage 3 or 4) is variable.^34^ It is noteworthy that our study included patients from Southern Africa, where diagnostic delays in TB patients, especially those who are HIV-positive, are considerable.^39^^,^^40^ It is possible that some of our patients presented with a subacute pericarditis after pulmonary TB. In addition, patients with TB pericarditis may progress at different rates between stages or even skip stages and present rapidly with constriction depending on the immune response.^19^ These combined factors could explain the presence of more advanced stages of pericardial disease (stage 3 or 4, ie, constriction) in our cohort.

Chronic changes after pericardial inflammation may result in the development of fibrosis (with or without constrictive physiology) and pericardial calcification.^41^ Calcifications are therefore rarely present in the acute and subacute stages of pericarditis and are generally absent at the time of diagnosis of TB pericarditis in sub-Saharan Africa.^7^ This is consistent with our results at baseline and after anti-TB treatment, with a low and stable rate of pericardial calcifications (2% vs 1%, *P* = 0.4). Cardiac CT may be useful as a complementary modality to assess pericardial thickening and pericardial calcifications and allows for preoperative planning.^41^ However, a recent study (including 17.3% of TB-related pericarditis) showed that pericardial calcification was not associated with adverse outcomes after pericardiectomy in chronic constrictive pericarditis.^42^

Tuberculous constrictive pericarditis can persist for many years and eventually lead to diastolic heart failure secondary to a noncompliant pericardium.^8^^,^^30^^,^^43^ Left untreated, TB constrictive pericarditis can lead to recurrent acute heart failure hospitalization and, in some cases, death.^8^ Data from the first decade of the millennium in sub-Saharan Africa indicate the seriousness of the problem: following initial diagnosis of TB pericarditis, more than one in 4 patients died within 6 months and mortality rose to 40% in patients with HIV coinfection who were not receiving ART.^5^ Standard, four-drug anti-TB therapy for 6 months is the treatment mainstay that significantly reduces mortality and hospitalization, especially in patients with HIV coinfection.^8^^,^^44^ However, progression to TB constrictive pericarditis, even with optimal anti-TB therapy, has been reported in to 20% to 30% of cases.^12^^,^^43^ Whether corticosteroids are effective in reducing mortality or progression to constriction is not clear.^7^^,^^43^^,^^45^ According to the European Society of Cardiology pericardial disease guidelines, it may be reasonable to use adjunctive corticosteroids in patients with TB pericarditis without HIV, and to avoid corticosteroids in individuals with HIV because of the increased risk of malignancy.^44^ Because the analysis of TTEs was done centrally, we could not intervene in the clinical management of patients. Pericardiotomy remains the appropriate treatment for established TB constrictive pericarditis and is recommended if the patient’s condition is not improving or is deteriorating after 4 to 8 weeks of appropriate TB therapy.^44^

Outcomes of pericardial abnormalities improved in our study, with significantly less pericardial effusion and pericardial thickening after treatment. However, given the limited number of TTEs 6 months after anti-TB treatment, additional data, including follow-up several years after end of anti-TB treatment, are required to assess long-term outcomes such as TB constrictive pericarditis and heart failure. Early diagnosis of TB pericarditis and early treatment to reduce morbidity and mortality are public health issues in endemic regions where it could be possible to reverse the course of this burdensome disease.^20^ A recent International Position Statement on multimodality cardiac imaging in pericardial diseases^41^ and current European Society of Cardiology guidelines recommend to perform a TTE (class I, level of evidence C) in all patients with suspected acute pericarditis and in all patients with suspected pericardial effusion.^44^ Given the high prevalence of pericardial effusion and signs of constriction in our cohort, routine TTE at baseline in all newly diagnosed TB patients could have prognostic and therapeutic implications in cases of moderate to severe effusion or already established constriction, although more long-term data are needed to establish such a recommendation.

Our study has several limitations. First, due to the limited resources and older echocardiography machines in Lusaka, Tissue Doppler Imaging was not always available, which limited the interpretation of diastolic function and constriction. However, the integration of other parameters (E/A, deceleration time, IVC diameter and respiratory variability, respiratory variability of mitral inflow E velocity, respirophasic septal shift or diastolic septal bounce, diastolic hepatic vein expiratory reversal/forward flow velocity) allowed us to assess some other isolated signs of constriction. Second, while echocardiography is the first-line imaging test in patients with suspected pericardial disease, advanced imaging techniques (CT and/or MRI) are recommended to enhance the sensitivity and specificity of diagnosing constrictive pericarditis.^41^^,^^44^ CT and MRI are second-level imaging techniques used to assess calcifications (CT), pericardial thickness (CT or MRI), and inflammation (MRI), the degree and extent of pericardial involvement (CT or MRI), as well as myocardial or aortic involvement (MRI). Although echo can offer a crude visual assessment of pericardial thickness, its accuracy is limited, particularly with inappropriate gain setting, and does not formally diagnose other forms of cardiovascular involvement in TB. Multimodal imaging is therefore an important cornerstone for the accurate diagnosis, prognosis and follow-up of pericardial and other forms of TB-related cardiovascular disease. However, advanced imaging techniques are not routinely available in lower-income countries.^44^ Third, despite echocardiographic signs of pericardial involvement associated with signs of systemic inflammation, we had no electrocardiogram (ECG) and could not incorporate clinical signs of pericarditis (eg, rubbing) or constriction (eg, Kussmaul's sign) into our diagnostic evaluation. Fourth, in our study of patients with pulmonary TB, the absence of microbiological evidence of simultaneous TB involvement of the pericardium, or confirmation of pericardial inflammation by pericardial puncture or advanced imaging modality, underscores the need for immunological, bacteriological studies, autopsy studies, and multimodality cardiac imaging ^5^ in this understudied population. However, the tools to conduct such studies are largely unavailable in the high TB burden settings in sub-Saharan Africa. Fifth, our study includes only urban or peri-urban populations, and may not be representative of rural areas which may have less access to ART and anti-TB treatment and patients with more advanced disease. Finally, although this is the first study to investigate prospectively the echocardiographic outcomes of TB, the number of included patients was modest. Nevertheless, the clinical investigations were detailed and standardized.

In conclusion, we found that cardiac involvement, particularly signs of pericarditis, is frequent among pulmonary TB patients at the initiation of treatment. Treatment was clearly associated with a decrease in pericardial abnormalities. Our study highlights that echocardiographic screening of TB patients facilitates early detection of cardiovascular involvement, which may allow for timely medical management and prevent complications such as constriction and heart failure.

Perspectives **COMPETENCY IN PATIENT CARE AND PROCEDURAL SKILLS:** Routine echocardiographic screening in newly diagnosed TB patients identified common pericardial abnormalities, and early stages of pericardial abnormalities largely showed regression after completion of an anti-TB treatment, enabling timely management.

**TRANSLATIONAL OUTLOOK:** Further research should explore long-term cardiovascular outcomes in TB patients, the benefits of routine echocardiographic screening in terms of clinical outcomes such as constriction and heart failure, and inform global health strategies for TB-related cardiovascular care.

## Funding support and author disclosures

Research reported in this publication was supported by the 10.13039/100000060National Institute of Allergy And Infectious Diseases of the 10.13039/100000002National Institutes of Health under Award Number U01AI069924 and the Swiss National Science Foundation (SNSF grant 32FP30-189498). The content is solely the responsibility of the authors and does not necessarily represent the official views of the National Institutes of Health or the SNSF. Dr Pilgrim reports research grants to the institution from Biotronik, Boston Scientific, Edwards Lifesciences, and consultancy/speaker fees from Biotronik, Edwards Lifesciences, Abbott, Biosensors, Medtronic, and HighLife. Dr Samim received funding for an online course from Edwards Lifesciences. All other authors have reported that they have no relationships relevant to the contents of this paper to disclose.

## Supplemental Data

Supplemental Materials

Video Clip 1

Video Clip 2

Video Clip 3

## References

[1] (2022). Global, regional, and National sex differences in the global burden of tuberculosis by HIV status, 1990-2019: results from the Global Burden of Disease Study 2019. Lancet Infect Dis.

[2] Obonyo N.G., Etyang A.O. (2023). Cardiovascular health priorities in sub-saharan Africa. SN Compr Clin Med.

[3] Minja N.W., Nakagaayi D., Aliku T. (2022). Cardiovascular diseases in Africa in the twenty-first century: gaps and priorities going forward. Front Cardiovasc Med.

[4] Keates A.K., Mocumbi A.O., Ntsekhe M., Sliwa K., Stewart S. (2017). Cardiovascular disease in Africa: epidemiological profile and challenges. Nat Rev Cardiol.

[5] van Heerden J.K., Louw E.H., Thienemann F., Engel M.E., Allwood B.W. (2024). The prevalence of pulmonary hypertension in post-tuberculosis and active tuberculosis populations: a systematic review and meta-analysis. Eur Respir Rev.

[6] Marcu D.T.M., Adam C.A., Mitu F. (2023). Cardiovascular involvement in tuberculosis: from pathophysiology to diagnosis and complications-A narrative review. Diagnostics.

[7] Mayosi B.M., Burgess L.J., Doubell A.F. (2005). Tuberculous pericarditis. Circulation.

[8] López-López J.P., Posada-Martínez E.L., Saldarriaga C. (2021). Tuberculosis and the heart. J Am Heart Assoc.

[9] Mayosi B.M. (2007). Contemporary trends in the epidemiology and management of cardiomyopathy and pericarditis in sub-Saharan Africa. Heart.

[10] Chammartin F., Dao Ostinelli C.H., Anastos K. (2020). International epidemiology databases to evaluate AIDS (IeDEA) in sub-Saharan Africa, 2012-2019. BMJ Open.

[11] Enane L.A., Duda S.N., Chanyachukul T. (2024). The tuberculosis sentinel research network (TB-SRN) of the international epidemiology databases to evaluate AIDS (IeDEA): protocol for a prospective cohort study in Africa, southeast Asia and Latin America. BMJ Open.

[12] Cremer P.C., Klein A.L., Imazio M. (2024). Diagnosis, risk stratification, and treatment of pericarditis: a review. JAMA.

[13] Welch T.D., Ling L.H., Espinosa R.E. (2014). Echocardiographic diagnosis of constrictive pericarditis: mayo Clinic criteria. Circ Cardiovasc Imaging.

[14] Nagueh S.F., Appleton C.P., Gillebert T.C. (2009). Recommendations for the evaluation of left ventricular diastolic function by echocardiography. J Am Soc Echocardiogr.

[15] Nagueh S.F., Smiseth O.A., Appleton C.P. (2016). Recommendations for the evaluation of left ventricular diastolic function by echocardiography: an update from the American society of echocardiography and the European association of cardiovascular imaging. J Am Soc Echocardiogr.

[16] van Buuren S., Groothuis-Oudshoorn K. (2011). Mice: multivariate imputation by chained equations in R. J Stat Software.

[17] Rubin D.B. (1987).

[18] Bourke C.D., Berkley J.A., Prendergast A.J. (2016). Immune dysfunction as a cause and consequence of malnutrition. Trends Immunol.

[19] Howlett P., Du Bruyn E., Morrison H. (2020). The immunopathogenesis of tuberculous pericarditis. Microb Infect.

[20] Isiguzo G., Du Bruyn E., Howlett P., Ntsekhe M. (2020). Diagnosis and management of tuberculous pericarditis: what is new?. Curr Cardiol Rep.

[21] Lazaros G., Vlachopoulos C., Lazarou E., Tsioufis K. (2021). New approaches to management of pericardial effusions. Curr Cardiol Rep.

[22] Sahiti F., Cejka V., Schmidbauer L. (2024). Prognostic utility of pericardial effusion in the general population: findings from the STAAB cohort study. J Am Heart Assoc.

[23] Savage D.D., Garrison R.J., Brand F. (1983). Prevalence and correlates of posterior extra echocardiographic spaces in a free-living population based sample (the Framingham study). Am J Cardiol.

[24] Krug S., Gupta M., Kumar P. (2023). Inhibition of host PARP1 contributes to the anti-inflammatory and antitubercular activity of pyrazinamide. Nat Commun.

[25] Rizvi F., Khan M., Jabeen A., Siddiqui H., Choudhary M.I. (2019). Studies on isoniazid derivatives through a medicinal chemistry approach for the identification of new inhibitors of urease and inflammatory markers. Sci Rep.

[26] Lind A., Reinsch N., Neuhaus K. (2011). Pericardial effusion of HIV-infected patients ? Results of a prospective multicenter cohort study in the era of antiretroviral therapy. Eur J Med Res.

[27] Ntsekhe M., Mayosi B.M. (2009). Cardiac manifestations of HIV infection: an African perspective. Nat Clin Pract Cardiovasc Med.

[28] Syed F.F., Sani M.U. (2013). Recent advances in HIV-associated cardiovascular diseases in Africa. Heart.

[29] Ntsekhe M., Wiysonge C.S., Gumedze F. (2008). HIV infection is associated with a lower incidence of constriction in presumed tuberculous pericarditis: a prospective observational study. PLoS One.

[30] Miranda W.R., Oh J.K. (2017). Constrictive pericarditis: a practical clinical approach. Prog Cardiovasc Dis.

[31] Syed F.F., Schaff H.V., Oh J.K. (2014). Constrictive pericarditis--a curable diastolic heart failure. Nat Rev Cardiol.

[32] Mayosi B.M., Wiysonge C.S., Ntsekhe M. (2006). Clinical characteristics and initial management of patients with tuberculous pericarditis in the HIV era: the Investigation of the Management of Pericarditis in Africa (IMPI Africa) registry. BMC Infect Dis.

[33] Collini V., Siega Vignut L., Angriman F., Braidotti G., De Biasio M., Imazio M. (2024). Age-stratified patterns in clinical presentation, treatment and outcomes in acute pericarditis: a retrospective cohort study. Heart.

[34] Imazio M., Brucato A., Maestroni S. (2011). Risk of constrictive pericarditis after acute pericarditis. Circulation.

[35] Frisancho-Kiss S., Coronado M.J., Frisancho J.A. (2009). Gonadectomy of male BALB/c mice increases Tim-3(+) alternatively activated M2 macrophages, Tim-3(+) T cells, Th2 cells and Treg in the heart during acute coxsackievirus-induced myocarditis. Brain Behav Immun.

[36] Li Z., Yue Y., Xiong S. (2013). Distinct Th17 inductions contribute to the gender bias in CVB3-induced myocarditis. Cardiovasc Pathol.

[37] Huber S.A. (2008). Coxsackievirus B3-induced myocarditis: infection of females during the estrus phase of the ovarian cycle leads to activation of T regulatory cells. Virology.

[38] Behr M.A., Edelstein P.H., Ramakrishnan L. (2018). Revisiting the timetable of tuberculosis. BMJ.

[39] Needham D.M., Foster S.D., Tomlinson G., Godfrey-Faussett P. (2001). Socio-economic, gender and health services factors affecting diagnostic delay for tuberculosis patients in urban Zambia. Trop Med Int Health.

[40] Kerkhoff A.D., Kagujje M., Nyangu S. (2021). Pathways to care and preferences for improving tuberculosis services among tuberculosis patients in Zambia: a discrete choice experiment. PLoS One.

[41] Klein A.L., Wang T.K.M., Cremer P.C. (2024). Pericardial diseases: international position statement on new concepts and advances in multimodality cardiac imaging. JACC Cardiovasc Imaging.

[42] Lee Y.H., Kim S.M., Kim E.K. (2023). Pattern of pericardial calcification determines mid-term postoperative outcomes after pericardiectomy in chronic constrictive pericarditis. Int J Cardiol.

[43] Chang S.A. (2017). Tuberculous and infectious pericarditis. Cardiol Clin.

[44] Adler Y., Charron P., Imazio M. (2015). 2015 ESC guidelines for the diagnosis and management of pericardial diseases: the task Force for the diagnosis and management of pericardial diseases of the European society of cardiology (ESC)Endorsed by: the European association for cardio-thoracic surgery (EACTS). Eur Heart J.

[45] Mayosi B.M., Ntsekhe M., Bosch J. (2014). Prednisolone and Mycobacterium indicus pranii in tuberculous pericarditis. N Engl J Med.

