# Supplementary material for: Cardiovascular Involvement in Tuberculosis Patients Treated in Southern Africa
Source: JACC Adv. 2024 Dec 5;4(1):101427. doi: 10.1016/j.jacadv.2024.101427 (PMC11667022; doi:10.1016/j.jacadv.2024.101427)
Supplement: Supplemental Materials [file mmc1.pdf]

Supplemental Table 1: Echocardiography results at TB diagnosis by HIV infection status. Categoricalvariables as positive count (% of total count) and continuous variables as median and interquartile range (IQR). Participants for whom echocardiography results could not be determined were excluded.

**Variable**	**Overall (N=286)**<br>n (%) / median (IQR)	**HIV-positive (N=109)**<br>n (%) / median (IQR)	**HIV-negative (N=177)**<br>n (%) / median (IQR)
**Abnormal LV geometry**	129 / 266 (48%)	57 / 102 (56%)	72 / 164 (44%)
concentric remodeling	112 (87%)	50 (88%)	62 (86%)
concentric hypertrophy	11 (9%)	4 (7%)	7 (10%)
eccentric hypertrophy	6 (5%)	3 (5%)	3 (4%)
**Aortic root dilatation**	3 / 241 (1%)	0 / 90 (0)	3 / 151 (2%)
**Ascending aorta dilatation**	3 / 230 (1%)	2 / 92 (2%)	1 / 138 (1%)
**Relevant aortic valve disease**	4 / 270 (1%)	4 / 107 (4%)	0 / 163 (0)
Aortic stenosis	0 / 4 (0)	0 / 4 (0)	0 / 0 (0)
Aortic regurgitation	4 / 4 (100%)	4 / 4 (100%)	0 / 0 (0)
**Relevant mitral valve disease**	3 / 270 (1%)	1 / 105 (1%)	2 / 165 (1%)
Mitral stenosis	0 / 3 (0)	0 / 1 (0)	0 / 2 (0)
Mitral regurgitation	0 / 3 (0)	0 / 1 (0)	0 / 2 (0)
**Relevant tricuspid valve disease**	2 / 266 (1%)	1 / 102 (1%)	1 / 164 (1%)
Tricuspid stenosis	0 / 2 (0)	0 / 1 (0)	0 / 1 (0)
Tricuspid regurgitation	2 / 2 (100%)	1 / 1 (100%)	1 / 1 (100%)
**LVEF** (%, visually assessed)	60.0 (55.0 – 65.0)	60.0 (55.0-65.0)	65.0 (60.0-65.0)
**LVEF** (%, Simpson BP)	60.3 (56.3 – 65.0)	59.5 (55.7-63.7)	60.8 (57.7-65.0)
**LV systolic dysfunction**	3 / 283 (1%)	2 / 108 (2%)	1 / 175 (1%)
**Diastolic dysfunction**	8 / 86 (9%)	6 / 33 (18%)	2 / 53 (4%)
**LV dilatation**	2 / 262 (1%)	0 / 98 (0)	2 / 164 (1%)
**LA dilatation**	44 / 266 (17%)	19 / 106 (18%)	25 / 160 (16%)
**RV dilatation**	15 / 240 (6%)	5 / 92 (5%)	10 / 148 (7%)
**RA dilatation**	27 / 227 (12%)	14 / 89 (16%)	13 / 138 (9%)
**TAPSE** (mm)	22.3 (19.5-25.0)	21.9 (19.0-24.0)	22.6 (19.9-25.1)
**TV annulus DTI S'** (cm/sec)	13.0 (12.0-14.7)	13.8 (12.7-15.9)	13.0 (11.8-14.2)
**RV longitudinal dysfunction**	11 / 264 (4%)	8 / 101 (8%)	3 / 163 (2%)
**RV FAC** (%)	46.7 (42.1-51.5)	46.7 (42.2-51.6)	46.7 (42.1-51.5)
**RV global dysfunction**	0 / 227 (0)	0 / 89 (0)	0 / 138 (0)
**RV/RA Gradient** (mmHg)	21.1 (18.6 – 27.5)	20.6 (19.5 – 26.5)	23.0 (17.6-29.1)
**Pericardial effusion**	134 / 286 (47%)	55 / 109 (50%)	79 / 177 (45%)
Mild	109 (81%)	46 (84%)	63 (80%)
Moderate	22 (16%)	9 (16%)	13 (16%)
Large	3 (2%)	0 (0)	3 (4%)
**Pericardial thickening**	86 / 286 (30%)	38 / 109 (35%)	48 / 177 (27%)
**Pericardial calcification**	7 / 285 (2%)	2 / 109 (2%)	5 / 176 (3%)
**Signs of constrictions**	103 / 244 (42%)	38 / 91 (42%)	65 / 153 (42%)
**Definite diagnosis of constriction**	13 / 105 (12%)	5 / 43 (12%)	8 / 62 (13%)
**Estimated central venous pressure** (mmHg)	10 (5-15)	10 (9-15)	10 (5-10)

LV = left ventricular; LVEF = left ventricular ejection fraction; LA = left atrial; RV = right ventricular; RA = right atrial; TAPSE = tricuspid annular plane systolic excursion; TV annulus DTI S' = derived tricuspid lateral annular systolic velocity wave S'; FAC = fractional area change

Supplemental Table 2: Echocardiography results at anti-TB treatment end by HIV infection status. Categorical variables as positive count (% of total count) and continuous variables as medianand interquartile range (IQR). Participants for whom echocardiography results could not be determined were excluded.

**Variable**	**Overall (N=105)**<br>n (%) / median (IQR)	**HIV-positive (N=43)**<br>n (%) / median (IQR)	**HIV-negative (N=62)**<br>n (%) / median (IQR)
**Abnormal LV geometry**	45 / 87 (52%)	22 / 35 (63%)	23 / 52 (44%)
concentric remodeling	43 (96%)	21 (95%)	22 (96%)
concentric hypertrophy	2 (4%)	1 (5%)	1 (4%)
eccentric hypertrophy	0 (0)	0 (0)	0 (0)
**Aortic root dilatation**	0 / 86 (0)	0 / 39 (0)	0 / 47 (0)
**Ascending aorta dilatation**	1 / 84 (1%)	1 / 37 (3%)	0 / 47 (0)
**Relevant aortic valve disease**	0 / 90 (0)	0 / 38 (0)	0 / 52 (0)
Aortic stenosis	0 / 0 (0)	0 / 0 (0)	0 / 0 (0)
Aortic regurgitation	0 / 0 (0)	0 / 0 (0)	0 / 0 (0)
**Relevant mitral valve disease**	1 / 89 (1%)	0 / 36 (0)	1 / 53 (2%)
Mitral stenosis	0 / 1 (0)	0 / 0 (0)	0 / 1 (0)
Mitral regurgitation	1 / 1 (100%)	0 / 0 (0)	1 / 1 (100%)
**Relevant tricuspid valve disease**	0 / 83 (0)	0 / 35 (0)	0 / 48 (0)
Tricuspid stenosis	0 / 0 (0)	0 / 0 (0)	0 / 0 (0)
Tricuspid regurgitation	0 / 0 (0)	0 / 0 (0)	0 / 0 (0)
**LVEF** (%, visually assessed)	60.0 (55.0-65.0)	60.0 (55.0-65.0)	60.0 (55.0-65.0)
**LVEF** (%, Simpson BP)	59.5 (56.5-65.0)	59.1 (55.6-61.6)	60.1 (57. -65.4)
**LV systolic dysfunction**	1 / 103 (1%)	1 / 43 (2%)	0 / 60 (0)
**Diastolic dysfunction**	3 / 57 (5%)	3 / 23 (13%)	0 / 34 (0)
**LV dilatation**	0 / 86 (0)	0 / 35 (0)	0 / 51 (0)
**LA dilatation**	6 / 86 (7%)	5 / 36 (14%)	1 / 50 (2%)
**RV dilatation**	1 / 72 (1%)	1 / 25 (4%)	0 / 47 (0)
**RA dilatation**	7 / 71 (10%)	0 / 26 (0)	7 / 45 (16%)
**TAPSE (mm)**	23.6 (21.0-26.0)	23.6 (21.2-26.0)	23.6 (20.9-26.0)
**TV annulus DTI S’** (cm/sec)	12.7 (11.6-14.0)	13.0 (12.2-14.0)	12.5 (11.2-13.9)
**RV longitudinal dysfunction**	3 / 97 (3%)	1 / 40 (2%)	2 / 57 (4%)
**RV FAC** (%)	44.4 (38.4-48.1)	45.4 (39.8-49.3)	42.8 (37.9-47.5)
**RV global dysfunction**	1 / 68 (1%)	0 / 24 (0)	1 / 44 (2%)
**RV/RA Gradient** (mmHg)	22.6 (18.2 – 27.7)	24.5 (18.2 – 31.5)	22.2 (21.1 – 23.7)
**Pericardial effusion**	16 / 98 (16%)	8 / 41 (20%)	8 / 57 (14%)
Mild	14 (88%)	8 (100%)	6 (75%)
Moderate	2 (12%)	0 (0)	2 (25%)
Large	0 (0)	0 (0)	0 (0)
**Pericardial thickening**	15 / 102 (15%)	4 / 42 (10%)	11 / 60 (18%)
**Pericardial calcification**	1 / 100 (1%)	1 / 41 (2%)	0 / 59 (0)
**Signs of constrictions**	33 / 88 (38%)	12 / 39 (31%)	21 / 49 (43%)
**Definite diagnosis of constriction**	9 / 65 (14%)	2 / 28 (7%)	7 / 37 (19%)
**Est. central venous pressure** (mmHg)	10 (10-10)	10 (10-10)	10 (10-10)

LV = left ventricular; LVEF = left ventricular ejection fraction; LA = left atrial; RV = right ventricular; RA = right atrial; TAPSE = tricuspid annular plane systolic excursion; TV annulus DTI S’ = derived tricuspid lateral annular systolic velocity wave S’; FAC = fractional area change

Supplemental Table 3: Changes in pericardial pathologies between TB diagnosis and end of anti-TBtreatment in a complete case analysis and in an imputed data analysis of all patients.

Sample proportion (%) of patients with pericardial abnormalities (in % with 95%-confidence interval [CI] in brackets) at TB diagnosis and end of anti-TB treatment. The proportions are compared with a test for equal proportions. **Panel A**: Complete case analysis of all patients (N=286); **Panel B**: Imputed data analysis of all patients; **Panel C**: Complete case analysis of patients with follow-up visits (N=105); **Panel D**: Imputed data analysis of patients with follow-up visits.

Abnormality	At TB diagnosis	End of TB treatment	Comparison	
	Prop. (95%-CI)	Prop. (95%-CI)	Diff. (95%-CI)	p-value
**Panel A: Complete case analysis of all patients**				
Pericardial effusion	47 (41 – 53)	16 (9 – 24)	-31 (21 – 40)	<0.001
Pericardial thickening	30 (25 – 35)	15 (8 – 22)	-15 (7 – 24)	0.002
Pericardial calcification	2 (1 – 4)	1 (-1 – 3)	-1 (-1 – 4)	0.38
Signs of constriction	42 (36 – 48)	38 (27 – 48)	-5 (-7 – 17)	0.44
Definite diagnosis of constriction	12 (6 – 19)	14 (5 – 22)	1 (-12 – 9)	0.78
**Panel B: Imputed data analysis of all patients**				
Pericardial effusion	47 (41 – 53)	18 (11 – 29)	-28 (-39 – -18)	<0.001
Pericardial thickening	30 (25 – 36)	20 (12 – 30)	-10 (-21 – 0)	0.06
Pericardial calcification	2 (1 – 5)	1 (0 – 6)	-1 (-4 – -2)	0.37
Signs of constriction	41 (35 – 48)	39 (28 – 51)	-3 (-16 – 11)	0.70
Definite diagnosis of constriction	18 (11 – 29)	12 (6 – 22)	-6 (-18 – 5)	0.29
**Panel C: Complete case analysis of patients with follow-up visits**				
Pericardial effusion	58 (49 – 68)	16 (9 – 24)	-42 (30 – 54)	<0.001
Pericardial thickening	40 (31 – 49)	15 (8 – 22)	-25 (14 – 37)	<0.001
Pericardial calcification	5 (1 – 9)	1 (-1 – 3)	-4 (-1 – 8)	0.11
Signs of constriction	48 (37 – 59)	38 (27 – 48)	-11 (-4 – 26)	0.16
Definite diagnosis of constriction	31 (6 – 56)	14 (5 – 22)	-17 (-10 – 43)	0.14
**Panel D: Imputed data analysis of patients with follow-up visits**				
Pericardial effusion	58 (48 – 67)	17 (11 – 26)	-41 (-53 – -29)	<0.001
Pericardial thickening	40 (31 – 50)	15 (10 – 24)	-25 (-36 – -13)	<0.001
Pericardial calcification	5 (2 – 12)	1 (0 – 7)	-4 (-9 – 1)	0.10
Signs of constriction	45 (35 – 56)	36 (27 – 46)	-10 (-24 – 5)	0.19
Definite diagnosis of constriction	19 (12 – 30)	12 (6 – 22)	-7 (-19 – 5)	0.24

Supplemental Table 4: Association of patient characteristics with pericardial abnormalities at anti-TB diagnosis.

Characteristic		Univariate analysis		Multivariate analysis	
	n (%)	OR (95%-CI)	p-value	OR (95%-CI)	p-value
**Pericardial effusion**	**134 (47%)**				
Age>=25 (reference)	112 (84)				
Age<25	22 (16%)	1.48 (0.76 – 2.92)	0.25	1.74 (0.86 – 3.51)	0.12
Male (reference)	104 (78%)				
Female	30 (22%)	0.85 (0.49 – 1.48)	0.58	0.76 (0.42 – 1.35)	0.34
HIV-negative (reference)	79 (51%)				
HIV-positive	55 (41%)	1.26 (0.78 – 2.04)	0.35	1.39 (0.84 – 2.32)	0.20
New case (reference)	100 (75%)				
Relapse case	34 (25%)	1.15 (0.67 – 1.99)	0.61	1.15 (0.66 – 2.01)	0.62
**Pericardial thickening**	**86 (30%)**				
Age>=25 (reference)	74 (86%)				
Age<25	12 (14%)	0.88 (0.42 – 1.85)	0.74	1.06 (0.49 – 2.28)	0.89
Male (reference)	68 (79%)				
Female	18 (21%)	0.88 (0.47 – 1.65)	0.70	0.80 (0.42 – 1.53)	0.50
HIV-negative (reference)	48 (56%)				
HIV-positive	38 (44%)	1.62 (0.95 – 2.75)	0.07	1.67 (0.96 – 2.92)	0.07
New case (reference)	62 (72%)				
Relapse case	24 (28%)	1.21 (0.68 – 2.18)	0.52	1.10 (0.60 – 2.02)	0.76
**Signs of constriction**	**103 (42%)**				
Age>=25 (reference)	83 (81%)				
Age<25	20 (19%)	1.71 (0.83 – 3.52)	0.15	1.74 (0.82 – 3.68)	0.15
Male (reference)	77 (75%)				
Female	26 (25%)	1.17 (0.64 – 2.13)	0.61	1.09 (0.59 – 2.03)	0.78
HIV-negative (reference)	65 (63%)				
HIV-positive	38 (37%)	1.06 (0.62 – 1.82)	0.82	1.15 (0.65 – 2.02)	0.64
New case (reference)	79 (77%)				
Relapse case	24 (23%)	0.91 (0.49 – 1.67)	0.76	0.97 (0.52 – 1.81)	0.92

Supplemental Table 5: Association of patient characteristics with pericardial abnormalities at the endof anti-TB treatment.

Characteristic		Univariable analysis		Multivariable analysis	
	n (%)	OR (95%-CI)	p-value	OR (95%-CI)	p-value
**Pericardial effusion**	**16/98 (16%)**				
Age>=25 (reference)	13 (81%)				
Age<25	3 (19%)	1.23 (0.31 – 4.91)	0.77	2.05 (0.44 – 9.65)	0.36
Male (reference)	14 (88%)				
Female	2 (12%)	0.63 (0.13 – 3.07)	0.57	0.52 (0.09 – 3.00)	0.46
HIV-negative (reference)	8 (50%)				
HIV-positive	8 (50%)	1.48 (0.50 – 4.34)	0.48	1.50 (0.43 – 5.25)	0.53
New case (reference)	9 (56%)				
Relapse case	7 (44%)	2.44 (0.80 – 7.42)	0.12	2.39 (0.70 – 8.16)	0.17
**Pericardial thickening**	**15/102 (15%)**				
Age>=25 (reference)	13 (87%)				
Age<25	2 (13%)	0.79 (0.16 – 3.90)	0.77	0.65 (0.12 – 3.51)	0.62
Male (reference)	14 (93%)				
Female	1 (7%)	0.35 (0.04 – 2.85)	0.32	0.38 (0.04 – 3.64)	0.40
HIV-negative (reference)	11 (73%)				
HIV-positive	4 (27%)	0.50 (0.15 – 1.68)	0.26	0.75 (0.19 – 2.98)	0.68
New case (reference)	13 (87%)				
Relapse case	2 (13%)	0.33 (0.07 – 1.57)	0.16	0.32 (0.06 – 1.68)	0.18
**Signs of constriction**	**33/88 (38%)**				
Age>=25 (reference)	27 (82%)				
Age<25	6 (18%)	1.51 (0.46 – 4.99)	0.50	1.34 (0.37 – 4.83)	0.66
Male (reference)	29 (88)				
Female	4 (12%)	0.60 (0.17 – 2.09)	0.42	0.53 (0.13 – 2.14)	0.37
HIV-negative (reference)	21 (64%)				
HIV-positive	12 (36%)	0.62 (0.25 – 1.50)	0.29	0.96 (0.34 – 2.73)	0.94
New case (reference)	27 (82%)				
Relapse case	6 (18%)	0.38 (0.13 – 1.09)	0.07	0.39 (0.13 – 1.21)	0.10

Supplemental Figure 1: Timepoints of transthoracic echocardiography (TTE); TB, tuberculosis.

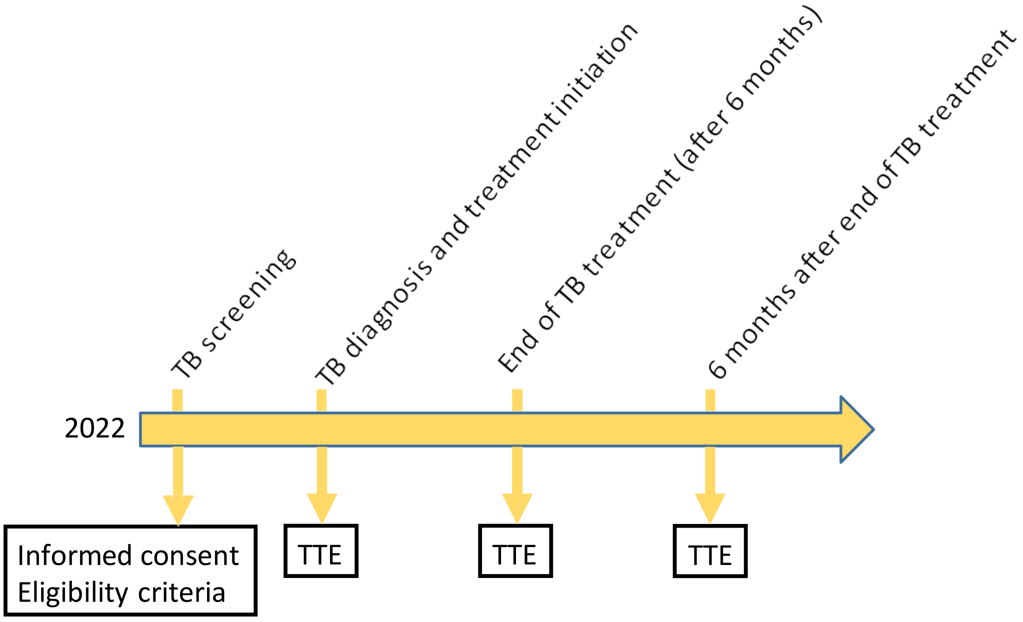

Supplemental Figure 2: Echocardiography diagnostic criteria algorithm for constrictive pericarditis.

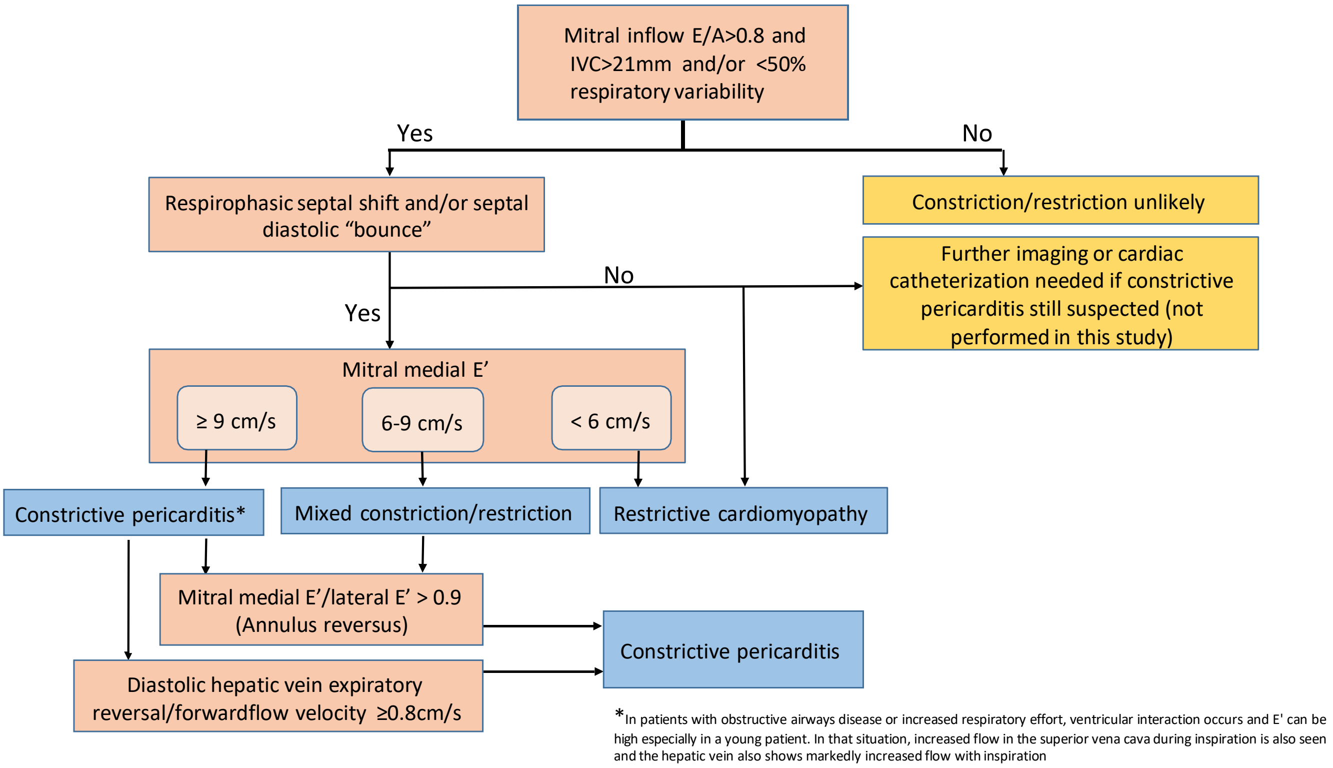

Supplemental Figure S3: Proportions of pericardial abnormalities at baseline and end of anti-TB treatment. Proportion of patients (%) with a pericardial effusion, thickening, or calcification, or signs of constriction or definite diagnosis of constriction at treatment start (286 patients) and end (105 patients).

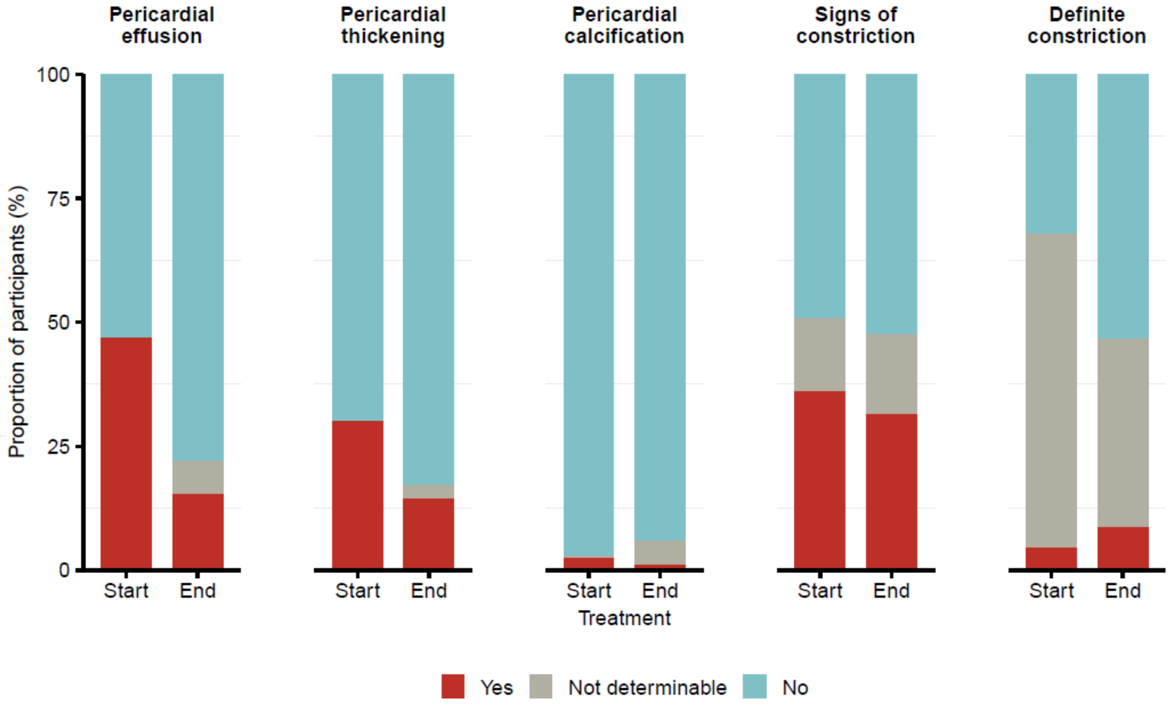

Acquisition Protocol[1]

The echocardiogram is performed with the subject in a steep left lateral position for parasternal and apical views. Subcostal views are obtained when the patient is supine.

At least three cardiac cycles of each view should be recorded during quiet respiration. If the heart rhythm is irregular (i.e. atrial fibrillation) at least five beats should be recorded.

Echo images in each view are recorded first with a depth and sector width that encompasses all the structures in the image plane and then at a depth and sector width optimized for the structures of interest. Gain and compression should be optimized for each view.

All clips need to be recorded with an ECG racing and clear calibration markings.

The following baseline parameters need to be documented

- Date, Time
- Patient name
- Blood pressure
- Height
- Weight

The echocardiographic examination should include the following standard views

- Parasternal Long Axis (PLAX)
- Right Ventricular Inflow View
- Parasternal Short Axis (PSAX)
- Right Ventricular Outflow View
- Apical Four-chamber View
- Apical Five-chamber View
- Apical Two-Chamber View
- Apical Long-Axis (3-Chamber) View
- Subcostal 4-Chamber View
- Subcostal Short axis view

Image acquisition

View	Clip/still frame	
Parasternal long axis view	1) 2D of on-axis LAX of the LV	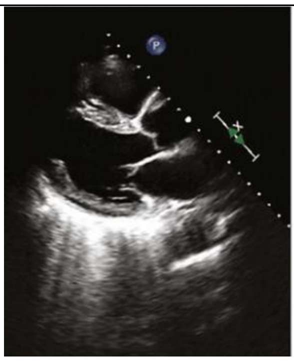 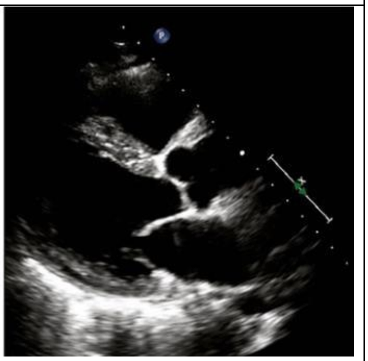
	2) LV M-mode perpendicular to the long axis of the LV	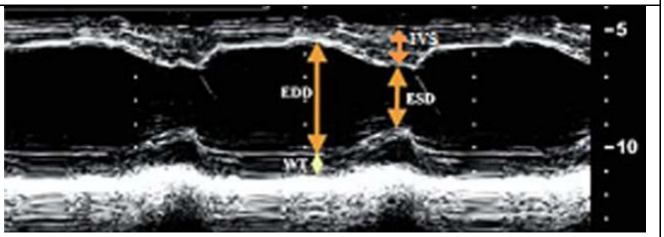
	3) Aortic valve M-mode tracing	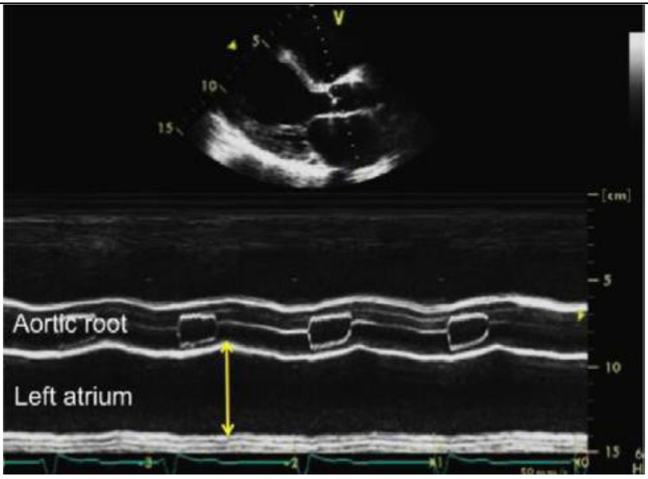

	2D zoom of aortic root	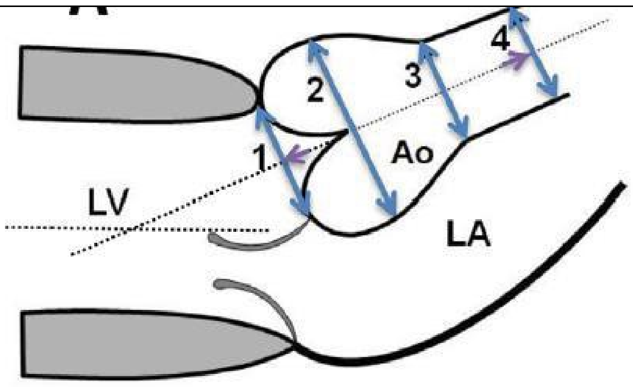
	2D zoom of Mitral valve	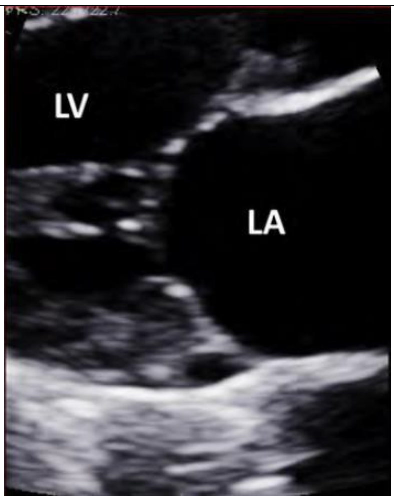
	Color Doppler of Mitral regurgitation	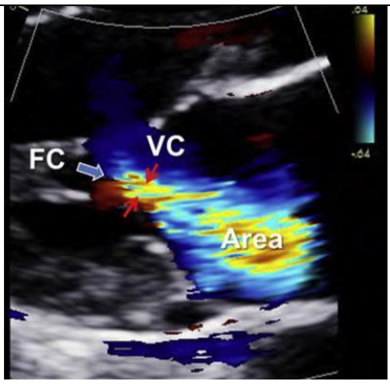
	Color Doppler of LVOT/aortic valve for Aortic regurgitation	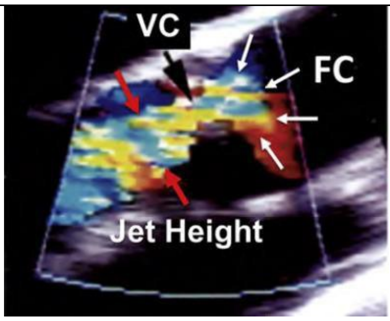

Right Ventricular Inflow View	2D of RA/TV/RV	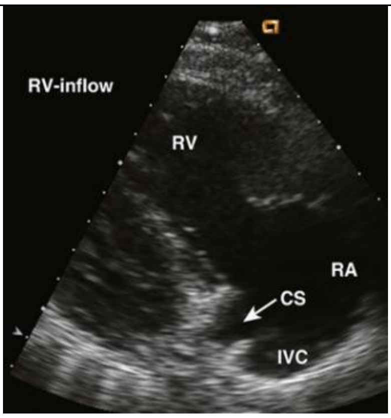
	Color Doppler of Tricuspid Regurgitation	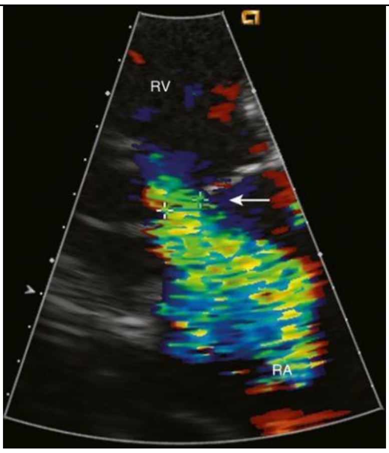
	CW Doppler of Tricuspid valve	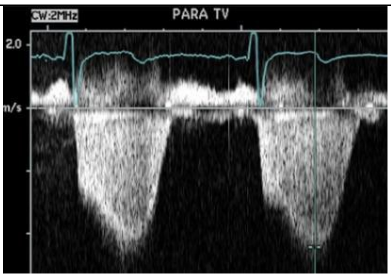
Parasternal Short Axis (PSAX)	2D mid-papillary muscle	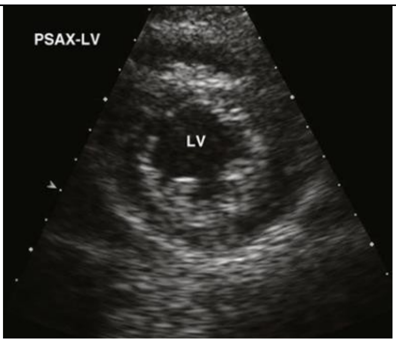

	2D mitral valve level	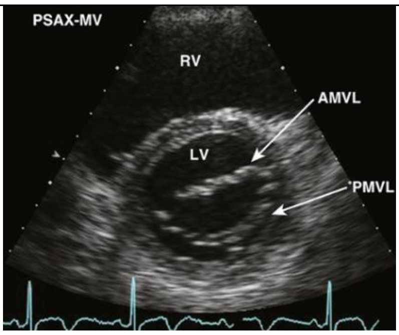
	2D zoom aortic valve	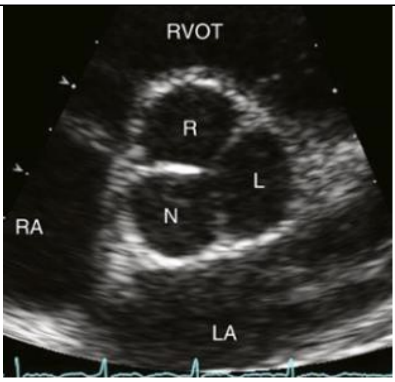
	Color Doppler aortic valve	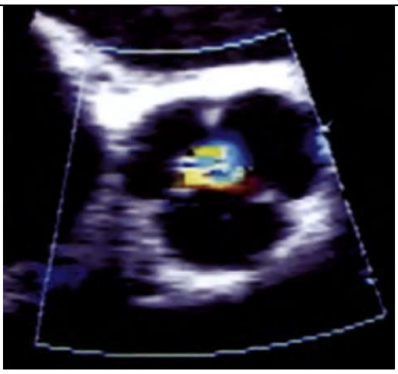
	2D zoom pulmonary valve	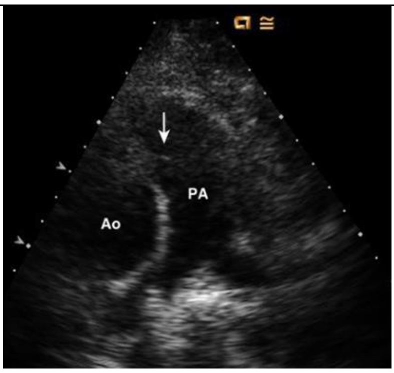

	Color Doppler pulmonary valve	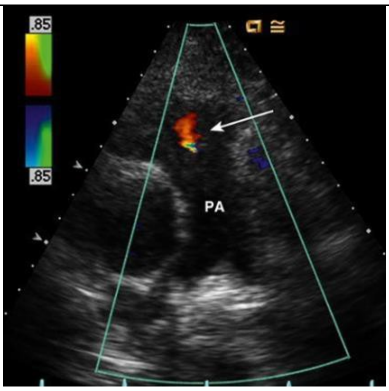
	PW Doppler pulmonary valve	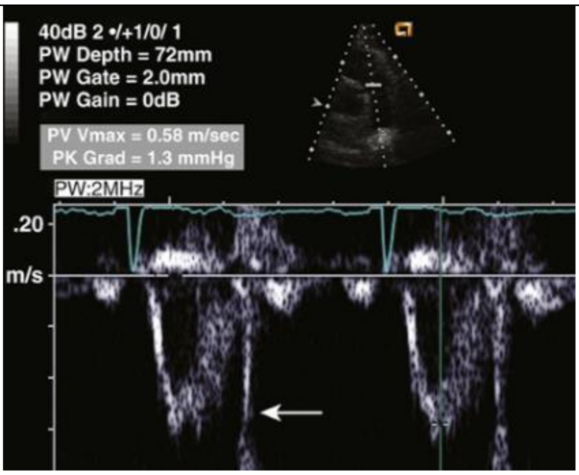
Right Ventricular Outflow View	2D of RVOT/pulmonary valve/Main pulmonary artery	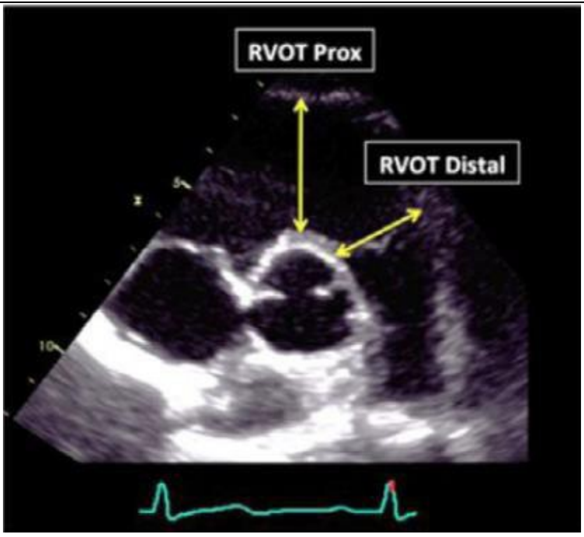
Apical Four-chamber View	2D view optimizing endocardial borders (required for GLS measurements)	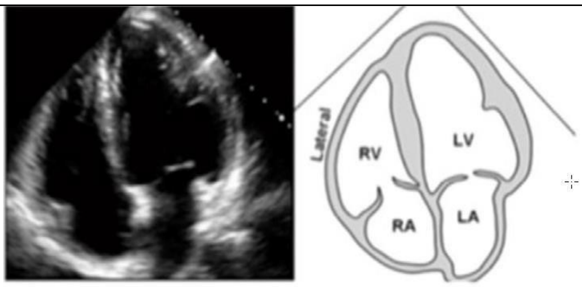

	2D view focusing on RA/RV	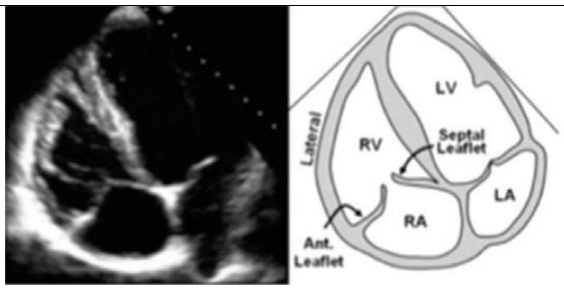
	Tricuspid Annular M-Mode (lateral) to measure TAPSE	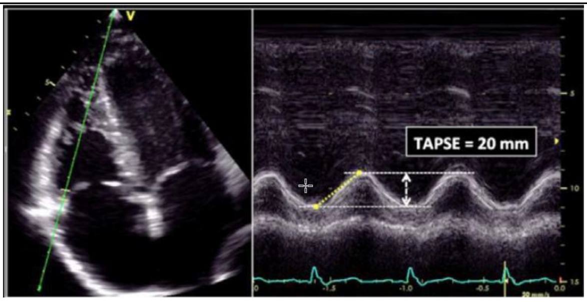
	Tricuspid Annular Tissue Doppler Systolic velocity	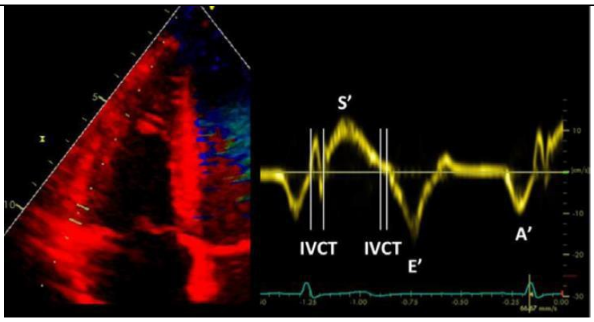
	2D zoom tricuspid valve	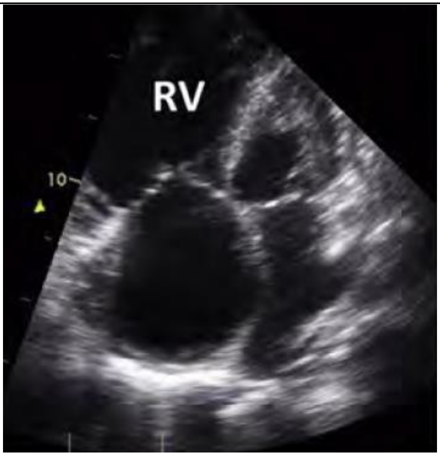
	Color Doppler tricuspid valve	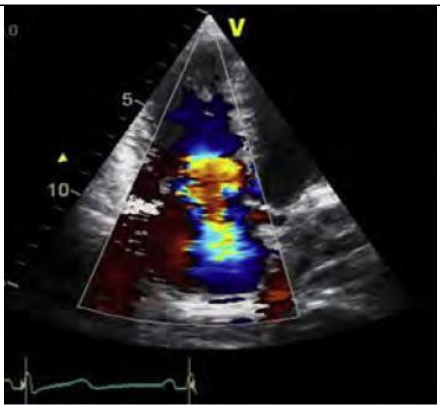

<table>
<tr><td></td><td>CW Doppler tricuspid valve</td><td>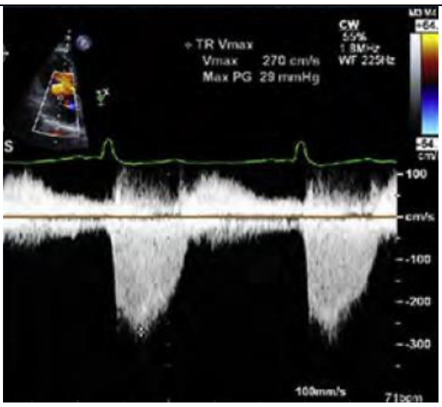
</td></tr>
<tr><td></td><td>2D zoom mitral valve</td><td>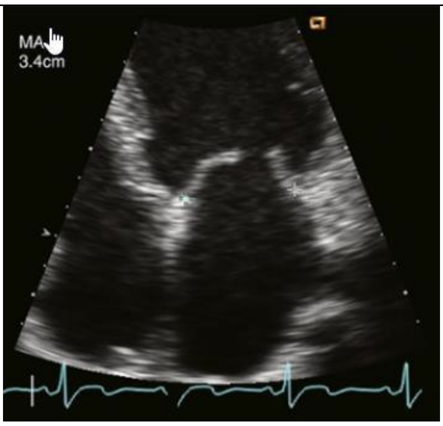
</td></tr>
<tr><td></td><td>Color Doppler mitral valve</td><td>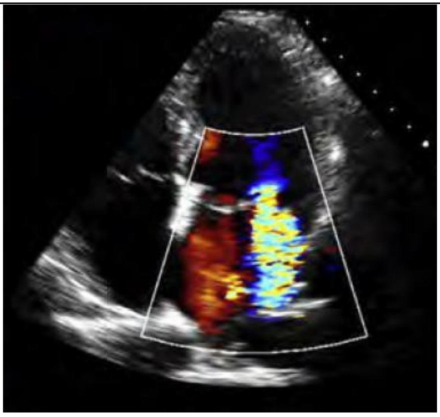</td></tr>
<tr><td></td><td>2D view focusing on LA/LV (for GLS)</td><td>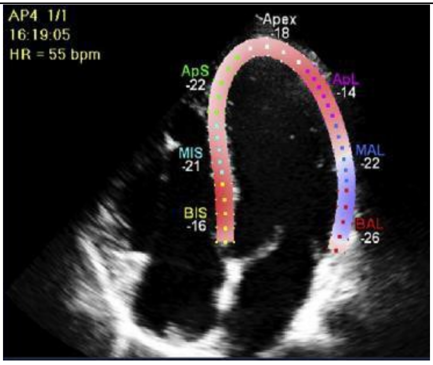
</td></tr>
</table>

	PW Doppler mitral valve (mitral inflow pattern)	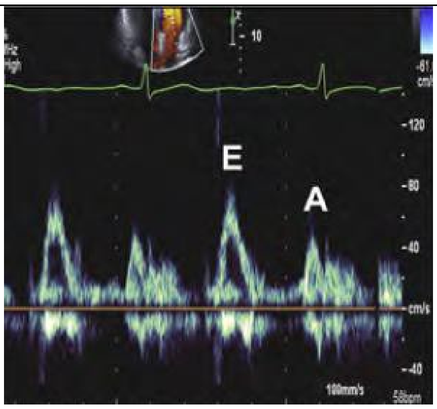
	Tissue Doppler myocardial velocities (septal, lateral side Mitral Annulus)	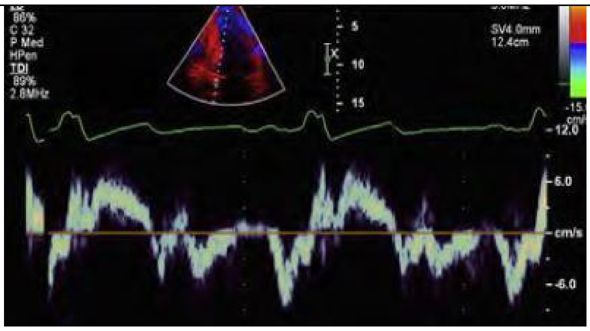 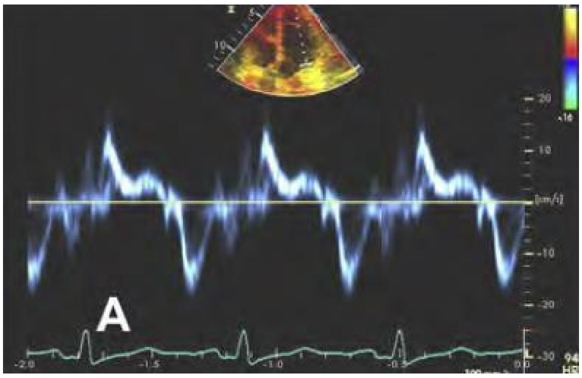

	2D view with decreased depth focusing on LV (for GLS)	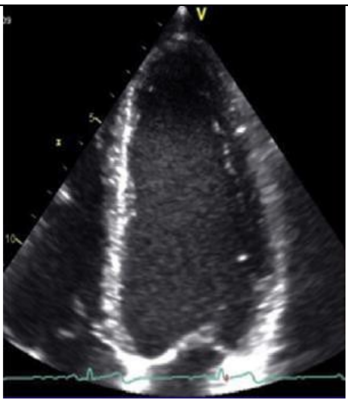 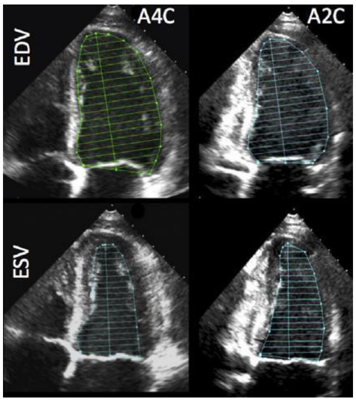
Apical Five-chamber View	Color Doppler aortic valve	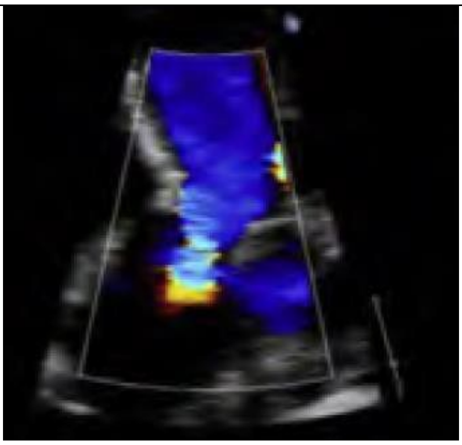
	CW Doppler aortic valve	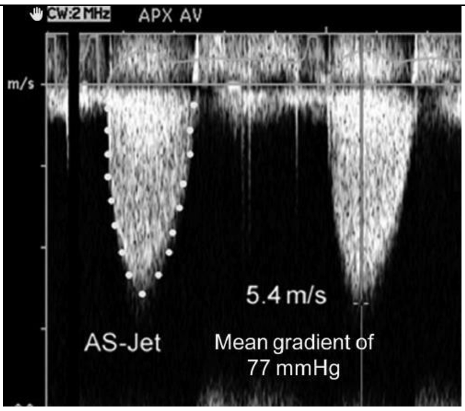

	PW Doppler LVOT	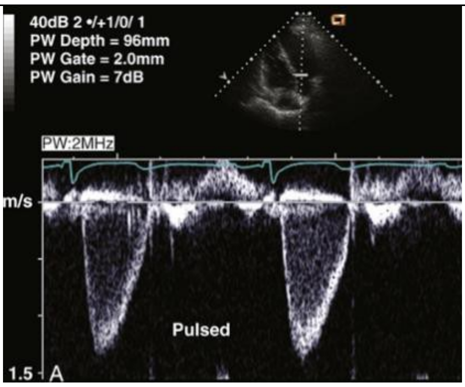
Apical Two-Chamber View	2D view optimizing endocardial borders (required for GLS measurements)	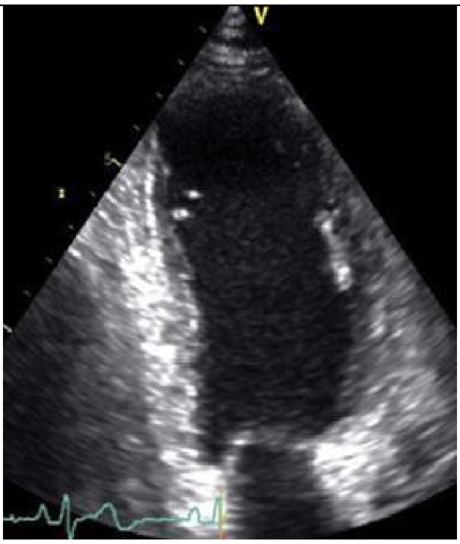
	Color Doppler mitral valve	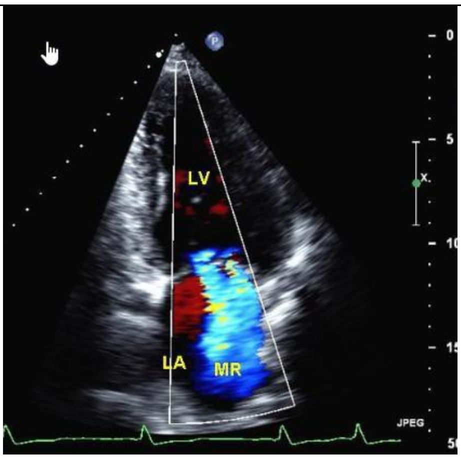

<table>
<tr>
<td>Apical Long-Axis (3-Chamber) View</td>
<td>2D view optimizing endocardial borders (required for GLS measurements)</td>
<td>
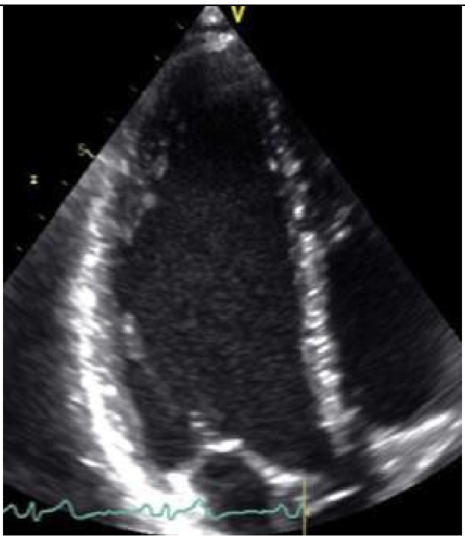

</td>
</tr>
<tr>
<td></td>
<td>Color Doppler mitral valve / aortic valve</td>
<td>
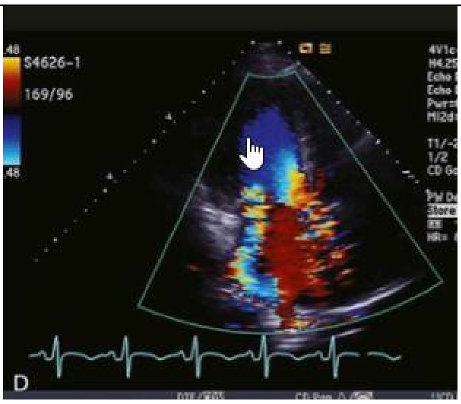

</td>
</tr>
<tr>
<td>Subcostal 4-Chamber View</td>
<td>2D optimizing endocardial borders of RV and LV</td>
<td>
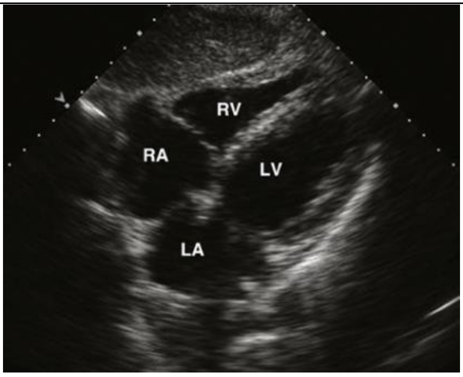

</td>
</tr>
<tr>
<td></td>
<td>Size and respiratory variation in the inferior vena cava</td>
<td>
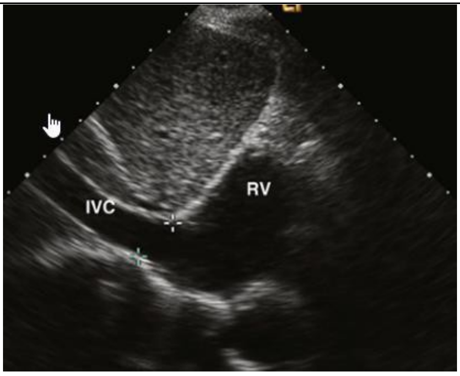

</td>
</tr>
</table>

Echocardiographic variables include left ventricular (LV) dimension (LVEDD, LVESD, IVSD, LVPWD in mm), LV geometry (according to LV RWT and LV mass index (g/m2)), LV systolic function (LV ejection fraction (LVEF in %)), LV diastolic function, Right ventricular (RV) dimension (RV base (mm)), RV systolic function (tricuspid annular plane excursion (TAPSE in mm), RV systolic-tissue Doppler imaging (S-TDI in cm/sec), Fractional area change (FAC in %)), Aortic valve assessment, Mitral valve assessment, Tricuspid valve assessment, Pulmonic valve assessment, Left atrial (LA) size (LA end-systolic diameter in parasternal long axis (mm) and LA volume biplane index (ml/m2)), Right atrial (RA) size (RA end-systolic area in cm2), Estimated pulmonary artery systolic pressure according (sPAP in mmHg), Estimated central venous pressure (inferior vena cava (IVC) diameter and respiratory variability), Pericardial effusion/thickening/calcification, aortic root (annulus, sinus of valsalva, sinotubular junction) and ascending aorta dimensions (mm).

Left ventricular (LV) dilatation was defined as LVEDD>58mm in men and 52mm in women, LV geometry was defined according to 2015 American Society of Echocardiography (ASE) and European Association of Cardiovascular Imaging (EACVI) recommendations.[2] Left ventricular (LV) systolic function was defined as LVEF<50% (according to Simpson Biplan or visual assessment). LV diastolic function was assessed according to 2016 ASE/EACVI guidelines.[3] Right ventricular (RV) dilatation was defined as RV base >41mm. Right ventricular longitudinal dysfunction was defined as TAPSE <17mm or S-TDI <9.5cm/sec; RV global dysfunction was defined as FAC<35%. Diastolic function was assessed and classified according to ASE/EACVI guidelines.[3] Inferior vena cava (IVC) normal diameter is <20mm; Normal RV/RA gradient is <30mmHg; Systolic pulmonary artery pressure (sPAP) was estimated by adding the estimated central venous pressure (CVP) to the RV/RA Gradient (SPAP = CVP + sPAP); PHT was defined as estimated sPAP> 40mmHg. Dimensions of aortic root (sinus of valsalva) and ascending aorta were assessed according to age and BSA separately for men and women.[4]

REFERENCES

1. Pilgrim T, Kalesan B, Karki P, Basnet A, Meier B, Urban P, et al. Protocol for a population-based study of rheumatic heart disease prevalence and cardiovascular outcomes among schoolchildren in Nepal. BMJ Open. 2012;2(3).

2. Lang RM, Badano LP, Mor-Avi V, et al. Recommendations for cardiac chamber quantification by echocardiography in adults: an update from the American Society of Echocardiography and the European Association of Cardiovascular Imaging. J Am Soc Echocardiogr. 2015;28(1):1–39.e14. Q8

3. Nagueh SF, Smiseth OA, Appleton CP, et al. Recommendations for the evaluation of left

ventricular diastolic function by echocardiography: an update from the American society of echocardiography and the European association of cardiovascular imaging. J Am Soc Echocardiogr. 2016;29(4):277–314.
4. Biaggi P, Matthews F, Braun J, Rousson V, Kaufmann PA, Jenni R. Gender, age, and body surface area are the major determinants of ascending aorta dimensions in subjects with apparently normal echocardiograms. J Am Soc Echocardiogr. 2009;22(6):720–725.
